# Comparison of scores for bimodality of gene expression distributions and genome-wide evaluation of the prognostic relevance of high-scoring genes

**DOI:** 10.1186/1471-2105-11-276

**Published:** 2010-05-25

**Authors:** Birte Hellwig, Jan G Hengstler, Marcus Schmidt, Mathias C Gehrmann, Wiebke Schormann, Jörg Rahnenführer

**Affiliations:** 1 Department of Statistics, TU Dortmund University, 44221 Dortmund, Germany; 2 Leibniz Research Centre for Working Environment and Human Factors, TU Dortmund University, Dortmund, Germany; 3 Department of Obstetrics and Gynecology, Johannes Gutenberg University, Medical School, Mainz, Germany; 4 Siemens Healthcare Diagnostics Products GmbH, Cologne, Germany

## Abstract

**Background:**

A major goal of the analysis of high-dimensional RNA expression data from tumor tissue is to identify prognostic signatures for discriminating patient subgroups. For this purpose genome-wide identification of bimodally expressed genes from gene array data is relevant because distinguishability of high and low expression groups is easier compared to genes with unimodal expression distributions.

Recently, several methods for the identification of genes with bimodal distributions have been introduced. A straightforward approach is to cluster the expression values and score the distance between the two distributions. Other scores directly measure properties of the distribution. The kurtosis, e.g., measures divergence from a normal distribution. An alternative is the outlier-sum statistic that identifies genes with extremely high or low expression values in a subset of the samples.

**Results:**

We compare and discuss scores for bimodality for expression data. For the genome-wide identification of bimodal genes we apply all scores to expression data from 194 patients with node-negative breast cancer. Further, we present the first comprehensive genome-wide evaluation of the prognostic relevance of bimodal genes. We first rank genes according to bimodality scores and define two patient subgroups based on expression values. Then we assess the prognostic significance of the top ranking bimodal genes by comparing the survival functions of the two patient subgroups. We also evaluate the global association between the bimodal shape of expression distributions and survival times with an enrichment type analysis.

Various cluster-based methods lead to a significant overrepresentation of prognostic genes. A striking result is obtained with the outlier-sum statistic (*p *< 10^-12^). Many genes with heavy tails generate subgroups of patients with different prognosis.

**Conclusions:**

Genes with high bimodality scores are promising candidates for defining prognostic patient subgroups from expression data. We discuss advantages and disadvantages of the different scores for prognostic purposes. The outlier-sum statistic may be particularly valuable for the identification of genes to be included in prognostic signatures. Among the genes identified as bimodal in the breast cancer data set several have not yet previously been recognized to be prognostic and bimodally expressed in breast cancer.

## Background

Genome-wide RNA expression analysis with microarrays has become a standard technology for screening for genes that are differentially expressed between different entities of tissues. In the clinical context, a frequent goal is the separation of patient groups with respect to prognosis or therapy outcome. The search for single genes as well as gene signatures with both prognostic power and corresponding biologically plausible interpretation remains a major challenge [1-3].

Identifying genes with discriminatory power using logistic regression type models often yields soft decision boundaries. However, the ideal situation for *personalized medicine *would be a sharp decision boundary, which corresponds to a clear separation of the expression values of a specific gene into two groups. This is particularly relevant for clinical routine, where usually not fresh frozen but formalin-fixed and paraffin-embedded tumor tissue is available. Since RNA from paraffin-embedded tissue is of lower quality than that of fresh frozen tissue the limited precision of quantitative analysis may add a further difficulty in classifying patients into low and high expression groups when genes with unimodal distributions are applied. In this situation bimodally distributed genes with a clear distinguishability between patients belonging to a high and a low expression group would mean an important practical advantage.

Bimodal distributions of RNA expression levels in tumor tissue may be caused by somatic mutations or by germline polymorphisms. A well-known example of a somatic or de novo mutation occurring during tumor development is amplification of the receptor tyrosine kinase proto-oncogene erbB2 which leads to increased RNA expression levels [2,4]. On the other hand also single nucleotide polymorphisms (SNPs) in the germ line have been shown to correlate with RNA expression in tumor tissue and may result in bimodal distribution curves [5]. Because of the clear distinguishability of high and low expression groups it could be an advantage to preferentially include bimodally distributed prognostic genes in prognostic signatures. In the literature various methods for the identification of genes with bimodal distributions have been introduced [6-9]. Several authors suggest approaches based on clustering the expression values of a gene into two groups and constructing scores for bimodality from the clustering result. Ertel and Tozeren (2008) [6] use the likelihood ratio of a normal model with one component and a normal mixture model with two components fitted to the expression values. The bimodality index suggested by Wang et al. (2009) [9] is based on the standardized difference of the cluster means. In this paper we introduce two further methods based on the comparison of the within cluster variance with the total variance.

The bimodality of a gene expression profile can be evaluated using the Dip Test of Unimodality suggested by Hartigan and Hartigan (1985) [7]. Other scores directly measure properties of the distribution, for example the kurtosis. In the case of two approximately equally sized groups the kurtosis is negative, distributions with many outliers have positive kurtosis. This approach was suggested by Teschendorff et al. (2006) [8].

An interesting alternative are outlier detection methods that search for genes with marked overexpression in a subset of cases [10-12]. The outlier-sum statistic explicitly identifies genes with extremely high or low expression values in a subset of the samples.

In this paper we present statistical methods for identifying prognostic genes whose expression values are clearly separated into two groups. Our approach is unsupervised in the sense that we first screen for genes with bimodal gene expression distributions and then evaluate their prognostic power. The resulting gene lists contain promising candidates for prognostic or predictive signatures. The clear separation into two groups makes the classification of future samples more reliable.

We first describe all measures and tests for the detection of bimodal distributions. Then we apply these measures to a microarray data set consisting of 194 breast cancer patients with corresponding whole genome RNA expression data. For each measure, we obtain a list of ranked genes. We evaluate the prognostic power of all genes with respect to disease-free survival using the logrank test for survival data. We discuss the results from two perspectives. First, we present a global assessment of the ability of the measures to identify prognostic genes. In an enrichment type analysis we quantify if significantly many genes with prognostic power are ranked high according to the bimodality measures. Second, we analyze the top-ranked genes in detail from a biological viewpoint, identifying concrete interesting new candidates.

## Methods

We first briefly present the *k-means *algorithm as a standard algorithm for clustering numerical values into two groups. We also describe a model-based clustering approach. Then we introduce different measures for assessing if the shape of a distribution supports the assumption that two clear subgroups can be identified. In other words, we present measures for testing bimodality of distributions. We briefly describe the *logrank *test for quantifying the statistical significance of survival time differences between two groups of patients, and we present the *Kolmogorov-Smirnov test *for the enrichment analysis comparing the prognostic genes with the genes with bimodal distributions.

### Cluster Algorithms

#### *K-means*

A straightforward method to assign patients to two groups based on their gene expression values is to use a clustering algorithm. One popular method is the *k-means* algorithm.

In the univariate case, let *x*_1_,..., *x*_*n *_be a set of observations. Given *k *<*n *initial distinct cluster centers the k-means algorithm partitions the set into *k *groups by minimizing the *Within Cluster Sum of Squares *, where *C*_*j*_, *j *= 1,..., *k*, is the *j*th cluster, *n*_*j *_is the number of elements in cluster *C*_*j *_and  is the cluster center. The algorithm is an iterative process that alternates between assigning data points to clusters based on their distances to cluster prototypes and updating the prototypes based on new cluster assignments.

K-means is sensitive to the initially randomly selected cluster centers [13]. As we want to discriminate a group with low expression values from a group with high expression values we choose for each gene its minimum and its maximum observed expression value as initial centers.

#### Model-based clustering

The assumption for this approach is that the distribution *Y *of a gene with bimodal expression can be expressed as a mixture of two gaussian distributions *Y*_1 _and *Y*_2 _with parameters *μ*_1_,  and *μ*_2_, , respectively.(1)

where Δ ∈ {0, 1} with *P*(Δ = 1) = *π*. *π *is the probability of belonging to *Y*_1_. Let *ϕ*_*θ *_denote the normal density with parameters *θ *= (*μ*, *σ*^2^). Then the density of *Y *is given by(2)

The parameters of the mixture model are determined using the EM-algorithm.

To fit the models to our data we make use of the R package mclust[14,15]. Typically equality of variances is not assumed and we fit models with unequal variances. However, in cases with only one outlier in the expression distribution a model with unequal variances is not suitable since one component has variance 0. In this case we fit a two component model with equal variances.

### Tests for bimodality

The first two scores for testing bimodality quantify whether the partitioning of the expression values into two groups is adequate. We want to quantify whether assuming two clusters is more appropriate than assuming one cluster. The tests are based on the decomposition of the *Total Sum of Squares * as(3)

where BSS is the *Between Cluster Sum of Squares *and WSS the *Within Cluster Sum of Squares*.

#### Variance reduction score

The *variance reduction score *(VRS) is defined as the ratio of WSS and TSS:(4)

VRS measures the proportion of variance reduction when splitting the data into two clusters. The value of this score lies in the interval [0; 1], and a low score indicates an informative split.

#### Weighted variance reduction score

The *weighted variance reduction score *(WVRS) measures the variance reduction independent of the cluster sizes. In the numerator we calculate the mean of the two within cluster variances. The denominator is the sample variance as above for the VRS. We define(5)

In this case the variance of a cluster with few observations has the same influence as the variance of a large cluster. The value of this score can be larger than 1. Again, a low score reflects bimodality. WVRS has the ability to also identify splits into two clusters with extremely unequal sample sizes.

#### Dip Test of Unimodality

The *Dip Test *of Unimodality was suggested by Hartigan and Hartigan (1985) [7]. The *dip statistic *is defined as the maximum difference between an empirical distribution function and the unimodal distribution function that minimizes that maximum difference.

For two arbitrary bounded functions *F *and *G *define *ρ*(*F*, *G*):= sup_*x*_|*F*(*x*)-*G*(*x*)|. Define  for a class  of bounded functions. Let  denote the class of unimodal distribution functions. Then the dip of a distribution function *F *is defined by . To test the null hypothesis that *F *has a unimodal density Hartigan and Hartigan proposed the statistic *D*(*F*_*n*_), where *F*_*n *_is the empirical distribution function of a random sample of size *n*. The distribution of *D*(*F*_*n*_) is compared with the distribution of *D(F)*, where *F *is the uniform distribution on [0; 1]. Hartigan and Hartigan [7] showed that the dip is asymptotically larger for the uniform distribution than for any other unimodal distribution.

To calculate the dip statistic we make use of the R package diptest [16], which also provides a table of empirical percentage points of the dip statistic *D*(*F*_*n*_) for some specified sample sizes *n*. This distribution can be used to calculate p-values for the dip test, where the null hypothesis is a unimodal distribution.

#### Kurtosis

Teschendorff et al. (2006) [8] proposed an approach to identify genes with bimodal density based on model-based clustering and *kurtosis*. The general approach is based on two steps. To select the optimal number of clusters and to find bimodal expression profiles a clustering algorithm is used together with a model selection criterion. Then the kurtosis can be used to rank the bimodal genes according to if they define major subgroups or outlier subgroups. Kurtosis is related to the fourth central moment and can be defined by(6)

Given a gene's expression values *x *= (*x*_1_,..., *x*_*n*_) an unbiased estimate for the kurtosis is given by(7)

A gaussian distribution has kurtosis *K *= 0, whereas most non-gaussian distributions have either *K *> 0 or *K *< 0. Specifically, a mixture of two approximately equal mass normal distributions must have negative kurtosis since the two modes on either side of the center of mass effectively flatten out the distribution. A mixture of two normal distributions with highly unequal mass must have positive kurtosis since the smaller distribution lengthens the tail of the more dominant normal distribution [8]. If there is an 80%-20% split of the samples into two groups, then the kurtosis is close to 0 [9]. Therefore biologically interesting genes might be missed.

We calculate the kurtosis for all genes without a prior feature selection step.

#### Likelihood Ratio

Using the *likelihood ratio *of a normal model and a mixture normal model to identify bimodal distributions was suggested by Ertel and Tozeren (2008) [6]. We fit a normal model and a mixture normal model to the expression values for each gene using the mclust package and calculate the likelihood ratio(8)

where *L*_1 _is the likelihood of the normal model with one component and *L*_2 _is the likelihood of a normal mixture model with two components with unequal variance.

Small ratios indicate that the distribution is unimodal, whereas large ratios suggest that the expression values are bimodally distributed.

#### Bimodality Index

The *Bimodality Index *introduced by Wang et al. (2009) [9] is a criterion to identify and rank bimodal signatures from gene expression data. It is assumed that the distribution of a gene with bimodal expression can be expressed as a mixture of two normal distributions with means *μ*_1 _and *μ*_2 _and equal standard deviation *σ*. The standardized distance *δ *between the two populations is given by(9)

For identifying genes with bimodal distribution the null hypothesis is *δ *= 0. Then the bimodality index (BI) is defined by(10)

where *π *is the proportion of observations in the first component.

For a given data set *δ *and *π *can be estimated using mclust. Then BI can be calculated using the estimated values. Larger values of BI correspond to bimodal distributions where the two components are easier to distinguish. Wang et al. recommend BI = 1.1 as a cutoff to select bimodally distributed genes.

#### Outlier-Sum Statistic

Another approach to group genes by expression values is based on the calculation of *outlier sums *as proposed by Tibshirani and Hastie (2007) [12]. This method is able to detect genes with unusually high or low expression in some but not all samples. Tibshirani and Hastie assume that the samples fall into a reference and a disease group. However, as we only consider tumor samples we modified the method for our purpose.

Let *med*_*i *_and *mad*_*i *_be the median and the median absolute deviation of the expression values of gene *i*. First, the expression values *x*_*ij *_of each gene are standardized as follows:(11)

Let *q*_*r*_(*i*) be the *r*th percentile of the  values and IQR(*i*) = *q*_75_(*i*) - *q*_25_(*i*) the interquartile range. All values smaller than IQR(*i*) - *q*_25_(*i*) or greater than IQR(*i*) + *q*_75_(*i*) are defined to be outliers. The outlier-sum statistic for positive outliers is defined as(12)

*W*_*i *_is large if there are many outliers in the data or few extreme outliers with high values, and *W*_*i *_is zero if there are no outliers. Analogously the outlier-statistic for negative outliers is defined as(13)

The outlier-sum statistic is the maximum of *W*_*i *_and *W'*_*i *_in absolute value.

For the survival analysis we need to split patients into two groups. We consider the outliers that are used to calculate the outlier sum as one group and all other observations as the other group.

### Testing for prognostic relevance

For each gene we compare the hazard rates of two patient subgroups obtained by the different methods. The goal is to determine whether the survival in the two groups differs. We test the hypothesis *H*_0_*: h*_1_(*t*) = *h*_2_(t), for all *t *≤ *τ*, against the alternative that the hazard rates differ for some *t *≤ *τ*. *τ *is the largest time at which both groups have at least one subject at risk. We make use of the *logrank test *which is a nonparametric test for right-censored data first proposed by Mantel (1966) [17].

Let *t*_*1 *_<*t*_2 _< ... <*t*_*D *_be the distinct death times in the pooled sample. Then *d*_*ij *_is the number of events in the *j*th sample out of *Y*_*ij *_individuals at risk at time *t*_*i*_, *j *= 1, 2, *i = *1,..., *D*. Let *d*_*i *_= *d*_*i*1 _+ *d*_*i*2 _and *Y*_*i *_= *Y*_*i*1 _+ *Y*_*i*2 _be the number of events and the number at risk in the combined sample at time *t*_*i*_. The test statistic *Z *of the logrank test is given by(14)

When the null hypothesis is true and the sample sizes in the two groups are similar, asymptotically *Z *has a standard normal distribution. In the case of highly unequal sample sizes, especially if one group contains less than five patients, the asymptotic distribution under the null hypothesis is not appropriate anymore [18,19]. Thus we do not make use of the normal distribution for obtaining significance values. Instead, we use a permutation test. We calculate the test statistic for 100.000 random permutations of the patients and determine the percentage of values more extreme than the observed value of the test statistic. Using k-means we always get two groups of patients. However, using the model-based clustering approach and the outlier sum approach it is possible that for some genes all patients are in the same group so that the logrank test can not be applied. As these genes rather have unimodal expression profiles we decided to set the corresponding p-value 1.

### Adjustment for multiple testing

We use the logrank test described in the previous Section to test for prognostic relevance for each of the genes. Here, we have to take the multiple testing problem into account. The significance level *α *of a hypothesis test controls the Type I error, i.e. the probability of rejecting a true null hypothesis. If *m *independent true hypotheses are tested simultaneously one would expect *m *· *α *p-values *p *<*α*. Therefore one has to adjust for multiple testing.

There are different correction methods. The *Bonferroni-Holm method *[20] controls the *Familywise Error Rate *(FWER), i.e. the probability of rejecting at least one true null hypothesis.

The method suggested by Benjamini and Hochberg (1995) [21] controls the *False Discovery Rate *(FDR), which is the expected proportion of falsely rejected hypotheses among all rejected hypotheses. This procedure is less conservative than the Bonferroni-Holm method and often more appropriate for high-dimensional microarray data.

Let *p*_(1)_, . . ., *p*_(*m*) _denote the ordered p-values of *m *hypothesis tests, i.e. *p*_(*i*) _≤ *p*_(*i*+1)_. Then the adjusted p-values, also referred to as *q-values*, can be calculated iteratively as follows(15)

where *q*_(*m*) _= *p*_(*m*)_.

### Analyzing gene lists for enrichment with prognostic genes

To determine whether there is an enrichment of the top-scoring bimodality genes with prognostic genes, we rank the genes based on the different bimodality scores. We then select the 250 genes with smallest p-values of the logrank test. These genes differ for the different approaches (i.e. k-means, model-based clustering, outlier sum). In this context, we call these genes prognostic genes.

An enrichment of the top-scoring bimodality genes with prognostic genes corresponds to small ranks of prognostic genes in the ranked list. We test the null hypothesis that the ranks of the prognostic genes are uniformly distributed among the bimodality-ranked genes with a one-sample *Kolmogorov-Smirnov test*, which is a nonparametric test of equality with a given one-dimensional reference function. The Kolmogorov-Smirnov statistic quantifies a distance between the empirical distribution function of the sample and the cumulative distribution function of the reference distribution.

We perform the one-sided Kolmogorov-Smirnov test for testing the hypothesis that the cumulative distribution function of the ranks of the prognostic genes is not greater than the distribution function of a uniform distribution. Let *F*_*n *_denote the empirical distribution function of the sample and *F *the distribution function of the reference distribution, then the test statistic is given by(16)

The null distribution of this statistic is calculated under the null hypothesis that the sample is drawn from the reference distribution. If *F *is continuous the distribution of  does not depend on *F *but just on the sample size *n*. For *n *> 40 approximative p-values can be derived from the asymptotical distribution of [22].

### Patients and gene array analysis

The Mainz study cohort consists of 194 node-negative breast cancer patients who were treated at the Department of Obstetrics and Gynecology of the Johannes Gutenberg University Mainz between the years 1988 and 1998 [1]. All patients underwent surgery and did not receive any systemic therapy in the adjuvant setting. Gene expression profiling of the patients' RNA was performed using the Affymetrix HG-U133A array, containing 22283 probe sets, and the GeneChip System [23]. The normalization of the raw data was done using RMA [24] from the Bioconductor [25] package affy[26]. The raw .cel files are deposited at the NCBI GEO data repository [27] with accession number GSE11121.

## Results

We analyze and compare distributions of bimodality measures. All methods presented in the methodology section are applied to the Mainz cohort study. For all bimodality measures we present the top-scoring genes and we analyze their ability to detect prognostic genes. We discuss and interpret commonalities and differences in the conclusions section.

### Correlation between bimodality scores

We calculated the seven scores for quantifying bimodality for all 22283 genes measured in the Mainz study. First, we analyze the global correlations between the measures. The results show that indeed a clear group structure exists which enables a classification of the measures for our application.

We point out that only the dip score is directly connected to a significance value since it is based on a test statistic with corresponding null distribution. The null distribution provides p-values for rejecting the hypothesis of unimodality. The empirical percentage point for sample size *n *= 200 and test level *α *= 0.05 is 0.037. For 8 genes the dip statistic is larger than 0.037 such that the null hypothesis of a unimodal density can be rejected. For the other bimodality scores no direct significance calculation resulting in p-values is available. For the bimodality index Wang et al. recommend the cutoff 1.1 which is exceeded by 1596 genes. In Table 1 and 2 we present the pairwise Pearson and Spearman correlation for the seven scores applied to all genes. Both methods show similar results. Here, the likelihood ratio is transformed to the logarithmic scale, which increases the Pearson correlation to other scores considerably due to the removal of outlier effects. Considerable correlation can be observed for various pairs of scores. The log-likelihood ratio and the outlier sum have the largest correlation (0.863). The only other correlations larger than 0.5 are observed pairwise between log-likelihood ratio, WVRS, and kurtosis. In Table 2 we present the same analysis replacing the Pearson correlation with the Spearman rank correlation. The correlation coefficient of WVRS and kurtosis is now the largest (0.865), and the measures log-likelihood ratio, WVRS, kurtosis, and outlier sum all have pairwise high rank correlations.

**Table 1:** Pearson correlation of the bimodality scores

	VRS	WVRS	dip	kurtosis	BI	log(LR)	OS
VRS	1.000	0.314	-0.079	0.172	-0.491	-0.173	-0.118
WVRS	0.314	1.000	-0.054	0.544	-0.062	0.548	0.398
dip	-0.079	-0.054	1.000	-0.039	0.013	-0.049	-0.109
kurtosis	0.172	0.544	-0.039	1.000	-0.174	0.661	0.407
BI	-0.491	-0.062	0.013	-0.174	1.000	0.244	0.334
log(LR)	-0.173	0.548	-0.049	0.661	0.244	1.000	0.863
OS	-0.118	0.398	-0.109	0.407	0.334	0.863	1.000

**Table 2:** Spearman correlation of the bimodality scores

	VRS	WVRS	dip	kurtosis	BI	log(LR)	OS
VRS	1.000	0.683	-0.086	0.613	-0.464	0.226	0.363
WVRS	0.683	1.000	-0.125	0.865	-0.008	0.681	0.752
dip	-0.086	-0.125	1.000	-0.077	-0.009	-0.070	-0.144
kurtosis	0.613	0.865	-0.077	1.000	-0.100	0.736	0.692
BI	-0.464	-0.008	-0.009	-0.100	1.000	0.437	0.311
LR	0.226	0.681	-0.070	0.736	0.437	1.000	0.786
OS	0.363	0.752	-0.144	0.692	0.311	0.786	1.000

**Figure 1:**
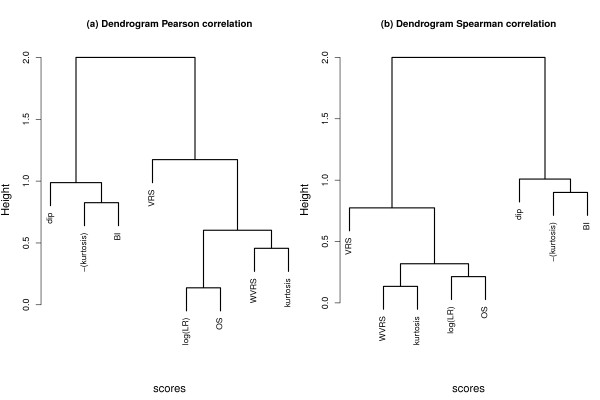
**Dendrograms of the correlation between the scores**. 1-correlation matrix of the scores was used as distance matrix for average linkage hierarchical clustering.

Figure 1 shows dendrograms generated using 1-*C *as distance matrix, where *C *is the correlation matrix of the seven scores. Both figures clearly show two groups. On the one side of the dendrogram there are the dip statistic, the bimodality index and -(kurtosis), i.e. the kurtosis score starting with largest values. Overall there is no large Pearson correlation between these scores, the distance is in most cases close to 1. The other group contains the highly correlated measures log-likelihood ratio, WVRS, kurtosis, and the outlier-sum statistic, as well as VRS which has a large distance to the other scores.

In summary, the outlier-sum statistic and the log-likelihood ratio have the highest correlation (Pearson 0.863 and Spearman 0.786). In both dendrograms these scores are thus very close. WVRS and kurtosis also belong to this group, whereas VRS, dip statistic, and bimodality index seem to measure different distributional features, at least according to the overall correlation.

**Figure 2:**
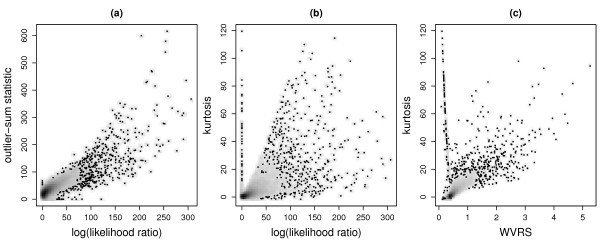
**Comparison of selected bimodality scores**. Pairwise comparisons of selected bimodality scores. Smoothed density representations of the scatterplots, obtained through kernel density estimation. The higher the local density is, the darker are the areas. The points represent the first 500 observations in the areas of lowest regional density. These observations can be regarded as potential outliers.

Figure 2 shows smoothed scatterplots for the comparison of selected scores. The strong positive correlation between the log-likelihood ratio and the outlier-sum statistic is clearly visible (Figure 2(a)). The areas of highest local density are at low values for the both scores. The correlation between the log-likelihood ratio and the kurtosis is smaller, but still obvious. However, at higher values of these scores there is a large dispersion; there are some genes with small log-likelihood ratio but very large positive kurtosis (Figure 2(b)). WVRS and the kurtosis are also positively correlated. Some of the genes with largest positive kurtosis have medium WVRS. The highest local density is at WVRS values between 0 and 1.

### Distributional shapes of genes with extreme bimodality scores

**Figure 3:**
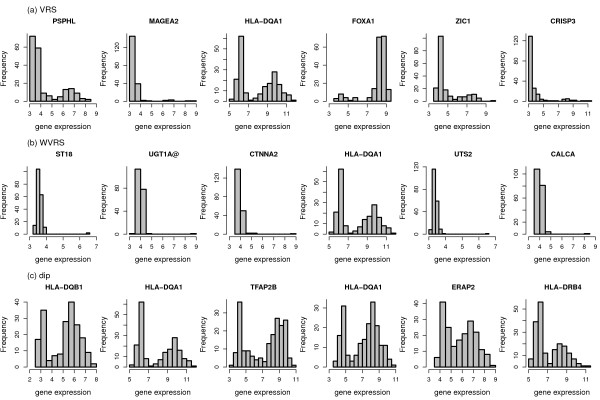
**Histograms of the top 6 genes for WVRS, VRS and dip**.

**Figure 4:**
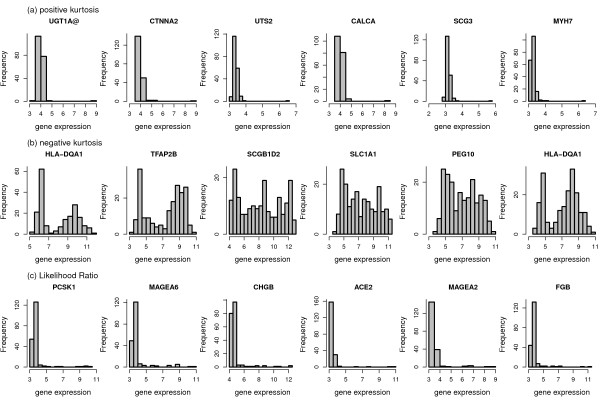
**Histograms of the top 6 genes for positive and negative Kurtosis and the likelihood ratio**.

**Figure 5:**
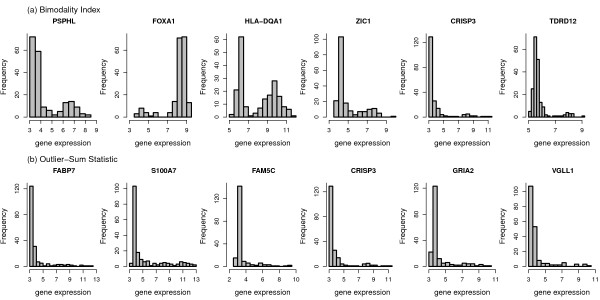
**Histograms of the top 6 genes for the bimodality index and the outlier sum**.

In order to obtain an impression of types of distributions detected by the different bimodality scores we present histograms of the expression values for the top 6 genes identified by each score, respectively (Figure 3, Figure 4, Figure 5). The most striking differences can be observed between the shapes detected by the correlated measures log-likelihood ratio, WVRS, kurtosis and outlier-sum statistic on the one side and all other scores on the other side. The former scores all identify genes with a main unimodal expression distribution and few additional outliers. The other scores detect mainly genes whose expression values can be grouped into two separated groups.

This difference can be exemplified with the clustering approaches resulting in the VRS and WVRS scores. The histograms for the top genes of VRS all indicate a large group and a smaller group (cf. Figure 3(a)). Except for FOXA1, the smaller group has larger expression values. On the other hand, the expression values of the top genes according to WVRS show a unimodal density with one or two outliers. Typically, most expression values of these genes are low, often in a range that could be regarded as noise, whereas the outliers have high expression values. This different behavior between VRS and WVRS is due to the larger impact of a subset with few samples when using WVRS, since then the variance reduction is calculated by averaging the variances of the two resulting subsets.

Similarly, the density of the genes with the largest positive kurtosis is unimodal with a small outlier group with high expression values (cf. Figure 4(a)), whereas the top 6 genes according to negative kurtosis show bimodal densities with almost equal size groups or a flat density (SCGB1D2). For the top genes according to the likelihood ratio one can see a large group and an outlier group with large variance. Also the outlier-sum statistic detects genes with densities with heavy tails in the higher intensities. For the dip statistic the top genes have clearly visible bimodal densities (Figure 3(c)). The genes with the largest bimodality index show bimodal densities, where the two groups can have unequal sizes (Figure 5(a)). There is not much overlap between the top six genes of all scores, except for the gene HLA-DQA1 with a clear bimodal histogram. The gene is among the top list for the scores detecting bimodal shapes, i.e. VRS, dip, negative kurtosis, and bimodality index, and additionally for WVRS.

In conclusion, all considered scores are applicable for the identification of genes with characteristic distributions. However, the distribution curves differ particularly with respect to the number of patients (samples) constituting the high expression group.

### Prognostic relevance of genes with extreme bimodality scores

**Figure 6:**
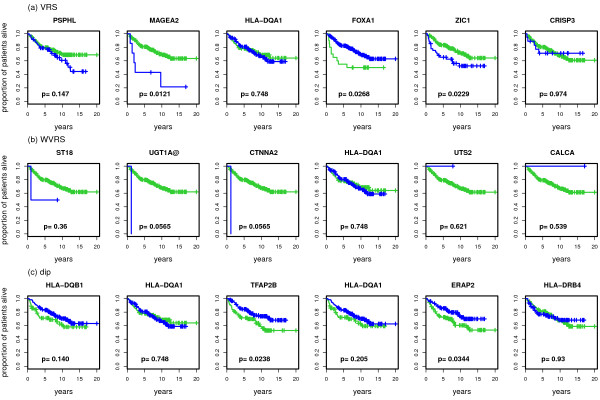
**Kaplan-Meier curves for top 6 genes for WVRS, VRS and dip**. Kaplan-Meier curves for the top 6 detected genes for WVRS, VRS and dip. In every graphic the green line denotes the survival in the group with lower expression values for the corresponding gene and the blue line the survival in the group with higher expression values. The graphics also contain the corresponding p-values of the logrank test.

**Figure 7:**
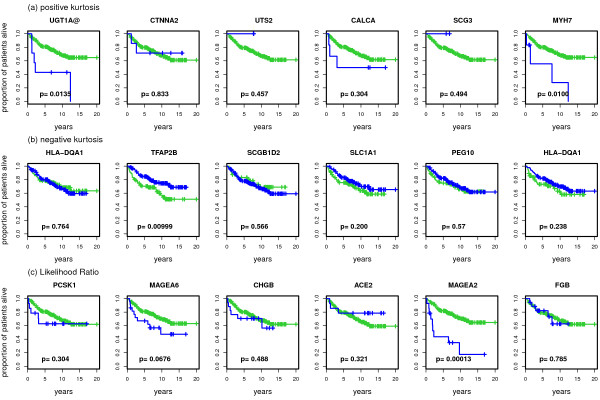
**Kaplan-Meier curves for the top 6 genes for positive and negative Kurtosis and the likelihood ratio**. Kaplan-Meier curves for the top 6 detected genes for positive and negative Kurtosis and the likelihood ratio. In every graphic the green line denotes the survival in the group with lower expression values for the corresponding gene and the blue line the survival in the group with higher expression values. The graphics also contain the corresponding p-values of the logrank test.

**Figure 8:**
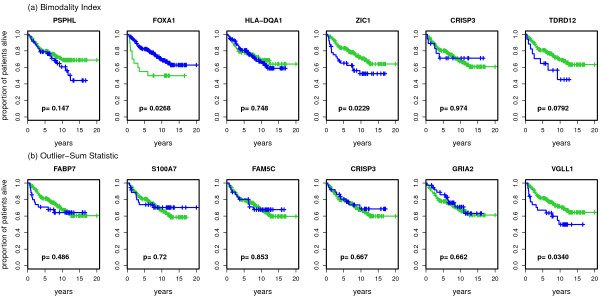
**Kaplan-Meier curves for the top 6 genes for the bimodality index and the outlier sum**. Kaplan-Meier curves for the top 6 detected genes for the bimodality index and the outlier sum. In every graphic the green line denotes the survival in the group with lower expression values for the corresponding gene and the blue line the survival in the group with higher expression values. The graphics also contain the corresponding p-values of the logrank test.

For the top genes presented in the previous section, we plot in Figure 6, Figure 7 and Figure 8 Kaplan-Meier curves for the top-scoring genes and corresponding p-values of the logrank test when discriminating patients according to low or high expression values. As described in the methodology section we use different methods to assign patients to a low and a high expression group. For VRS and WVRS we use the k-means algorithm, for the kurtosis based approach, the likelihood ratio, and the bimodality index we use the results of the model-based clustering, and for the outlier-sum statistic we use the main group and the outlier group. We argue that the k-means approach is also appropriate for the results of the dip test as we see that the k-means algorithm is able to separate the groups adequately at least for the top ranked genes.

For three of the top genes detected by VRS the survival in the two groups differs considerably. For MAGEA2 and ZIC1, the survival prediction is worse for the group with high expression values, for FOXA1 for the group with low expression values. The only patient in the high value group for UGT1A@ and CTNNA2 drops out at an early point, resulting in a small p-value (*p *= 0.057, see Figure 6(b)). This patient is the same for both genes. Among the top 6 genes for the dip statistic there are two with p-values smaller than 0.05, namely TFAP2B and ERAP2. For both genes the survival in the group with lower expression values is worse. For two of the 6 genes with largest positive kurtosis, UGT1A@ and MYH7, the survival in the patient subgroups differs significantly. Patients with high expression values have worse prognosis (Figure 7(a)). Among the top 6 genes for negative kurtosis, there is just one (TFAB2B) that splits the samples in two groups with significantly different prognosis. MAGEA2 and MAGEA6 are the two genes identified as bimodal by the likelihood ratio score whose p-values of the logrank test are small. Here, the p-value for MAGEA2 is highly significant (*p *< 0.001). Among the 6 top-scoring genes based on the bimodality index the two genes FOXA1 and ZIC1 lead to a prognostic split. Among the top 6 genes according to the outlier-sum statistic only VGLL1 generates a p-value smaller than 0.05.

### Prognostic relevance of bimodality scores on a genome-wide scale

In a next step we systematically analyzed the overlap between two different gene sets, namely genes identified by a score and genes associated with prognosis. The goal is to find out if among the bimodal genes identified by the different scores genes associated with prognosis are overrepresented. Table 3 shows the top 10 genes for each of the scores with corresponding p-values of the logrank test and the associated q-values.

**Table 3:** Top 10 genes for the bimodality scores

VRS			WVRS			dip		
gene	p	q	gene	p	q	gene	p	q
PSPHL	0.147	0.483	ST18	0.360	0.703	HLA-DQB1	0.140	0.473
MAGEA2	0.012	0.158	UGT1A@	0.056	0.311	HLA-DQA1	0.748	0.914
HLA-DQA1	0.748	0.914	CTNNA2	0.056	0.311	TFAP2B	0.024	0.216
FOXA1	0.027	0.225	HLA-DQA1	0.748	0.914	HLA-DQA1	0.205	0.560
ZIC1	0.023	0.213	UTS2	0.621	0.860	ERAP2	0.034	0.251
CRISP3	0.974	0.993	CALCA	0.539	0.821	HLA-DRB4	0.929	0.981
PCSK1	0.053	0.304	SCG3	0.622	0.878	NLRP2	0.162	0.506
DSG1	< 10^-3^	0.033	TFAP2B	0.024	0.216	IQGAP1	0.089	0.385
MAGEA4	< 10^-3^	0.028	MYH7	0.072	0.345	SAT1	0.712	0.901
UGT2B4	0.835	0.950	HAPLN1	0.072	0.345	DSCC1	0.072	0.346

**pos kurtosis**			**neg kurtosis**			**likelihood ratio**		
**gene**	**p**	**q**	**gene**	**p**	**q**	**gene**	**p**	**q**

UGT1A@	0.013	0.137	HLA-DQA1	0.764	0.913	PCSK1	0.304	0.622
CTNNA2	0.833	0.941	TFAP2B	0.010	0.120	MAGEA6	0.068	0.298
UTS2	0.457	0.742	SCGB1D2	0.566	0.807	CHGB	0.488	0.764
CALCA	0.304	0.622	SLC1A1	0.200	0.514	ACE2	0.321	0.636
SCG3	0.494	0.768	PEG10	0.570	0.811	MAGEA2	<10^-03^	0.015
MYH7	0.010	0.120	HLA-DQA1	0.238	0.557	FGB	0.785	0.921
HAPLN1	0.733	0.897	PSD3	0.067	0.297	DSG1	0.024	0.181
FGA	0.417	0.713	*unknown*	0.022	0.172	MSLN	0.138	0.423
HTR2C	0.016	0.148	ERAP2	0.303	0.622	UGT2B4	0.112	0.382
GLUD2	0.986	0.995	HLA-DQB1	0.243	0.561	CARTPT	0.382	0.691

**bimodality index**			**outlier sum**					
**gene**	**p**	**q**	**gene**	**p**	**q**			
			
PSPHL	0.147	0.449	FABP7	0.486	0.783			
FOXA1	0.027	0.189	S100A7	0.720	0.904			
HLA-DQA1	0.748	0.909	FAM5C	0.853	0.954			
ZIC1	0.023	0.175	CRISP3	0.667	0.884			
CRISP3	0.974	0.990	GRIA2	0.662	0.882			
TDRD12	0.079	0.332	VGLL1	0.034	0.224			
TFAP2B	0.024	0.178	MAGEA6	0.055	0.287			
MAGEA3	0.032	0.206	ALB	0.196	0.551			
UGT8	0.023	0.175	ASCL1	0.530	0.811			
S100A7	0.817	0.936	MAGEA3	0.151	0.489			

For each of the scores the table contains the 10 top-scoring genes. Also the p-values obtained by the logrank test and the corresponding q-values are given.

For the genome-wide evaluation, we first sorted the genes based on the bimodality scores. For VRS and WVRS we generated gene lists with smallest scores at the top. For the dip statistic, the bimodality index, the likelihood ratio and the outlier-sum statistic we generated decreasing lists. For the kurtosis we look at both the increasing as well as the decreasing list. We then selected the 250 genes with smallest p-values for survival differences. These gene lists are different for the different approaches.

The results of Fisher tests and Kolmogorov-Smirnov tests for the scores are displayed in Table 4. Plots corresponding to a Kolmogorov-Smirnov type test are shown in Additional file 1: Supplemental Figure 1. For VRS, among the top 200 genes there is just one of the 250 prognostic genes, but among the top 1000 genes there are 16. This number is significantly larger than expected under the null hypothesis of independence between bimodality score and prognostic relevance (*p *= 0.01). The Kolmogorov-Smirnov test rejects the null hypothesis that the ranks of the prognostic genes are uniformly distributed (*p *< 10^-7^). Thus there is a clear enrichment with prognostic genes in the top-scoring genes.

**Table 4:** Results of enrichment tests

	Fisher test				KS test
score	200 genes	p-value	1000 genes	p-value	p-value
VRS	1	0.896	16	0.099	< 10^-07^
WVRS	4	0.188	13	0.333	0.547
dip	2	0.659	9	0.795	0.749
pos kurtosis	2	0.659	17	0.059	0.003
neg kurtosis	2	0.659	8	0.878	0.988
LR	1	0.896	21	0.005	< 10^-12^
BI	0	1.000	22	0.002	< 10^-12^
outlier sum	0	1.000	26	< 10^-04^	< 10^-12^

For WVRS, among the 1000 top-scoring genes there are just 13 of the 250 prognostic genes (*p *= 0.333). Apparently there is no enrichment with prognostic genes on top of the list and the global Kolmogorov-Smirnov test can not reject (*p *= 0.547). However, many of the prognostic genes have large WVRS values (see Additional file 1). These genes have expression distributions with a main unimodal distribution and an additional outlier group with large variance. For the list based on the dip statistic just two of the prognostic genes are among the top 200 and 9 among the top 1000 genes. Here, the null hypothesis of the Kolmogorov-Smirnov test can not be rejected (*p *= 0.749).

Among the first 1000 genes on the list with decreasing kurtosis values there are 17 highly prognostic genes (*p *= 0.059), whereas on the increasing list there are 8 genes (*p *= 0.878). The result of the Kolmogorov-Smirnov test for the decreasing kurtosis list is borderline significant (*p *= 0.003). The prognostic genes are overrepresented among the first half of the ranked gene list (see Additional file 1). For the likelihood ratio the ranks of the prognostic genes are significantly not uniformly distributed (*p *< 10^-12^), and among the top 1000 genes we find 21 prognostic genes (*p *= 0.005). For the bimodality index the global enrichment is also highly significant (*p *< 10^-12^). There is a significant enrichment for the top-ranked genes. 22 of the 250 prognostic genes are among the first 1000 genes according to the bimodality index (*p *= 0.002).

Lastly, for the outlier-sum statistic a highly significant overrepresentation of the prognostic genes among the top-scored genes can be observed (*p *< 10^-12^). Among the first 1000 genes are 26 highly prognostic genes (*p *< 10^-4^).

This shows that on a global level the likelihood ratio, the bimodality index and the outlier-sum statistic work best for the identification of prognostic genes with bimodal density, many prognostic genes have densities with heavy tails. The clustering based approaches VRS is better suited to detect prognostic genes than the dip statistic.

It is important to note that we found that the threshold 250 for determining the prognostic genes is not critical. We tried various other cutoffs. Using 50 prognostic genes, no significant results are obtained. In the range from 100 to 1000 genes we found that the results for VRS, bimodality index, likelihood ratio and the outlier-sum statistic are similar.

### Validation on other data sets

To validate our results we repeated our analysis on an other free available data set. The raw .cel files and clinical parameters of the Rotterdam cohort [28,29] were downloaded from the NCBI GEO data repository with accession number GSE2034 (n = 286). Here, the outlier-sum statistic and the likelihood ratio also have the smallest p-values of the logrank test (see Additional file 2: Supplemental Figure 2). For the bimodality index there is also a significant enrichment (*p *= 0.01). However, we do not observe a significant overrepresentation of the prognostic genes among the top-scoring genes according to VRS. For the gene list based on decreasing kurtosis the we also find a significant enrichment (*p *< 10^-7^).

To determine whether our results also hold for known prognostic subgroups we used a pooled cohort of 766 patients from different free available data sets: GSE11121 (n = 200), GSE2034 (n = 286), GSE7390 (n = 177) [30] and GSE6532 (n = 103) [31,32]. We look at three subgroups defined by the expression of the two genes ESR1 and erbB2, namely ESR1+/erbB2- (n = 519), erbB2+ (n = 107) and ESR1-/erbB2- (n = 140). In this analysis the variable for assessing prognostic power of the genes is the distant metastasis free interval (MFI).

In the ESR1+/erbB2- subgroup of the pooled cohort the bimodality index has the smallest p-value of the logrank test (*p *< 10^-14^, see Additional file 3: Supplemental Figure 3). A significant enrichment of the top scoring genes with prognostic genes can also be observed for the likelihood ratio (*p *< 10^-8^) and for the outlier-sum statistic (*p *< 10^-5^). For the kurtosis score the prognostic genes have mainly negative values. There are also many genes with large positive kurtosis but these are not the top genes for positive kurtosis. For VRS the result of the Kolmogorov-Smirnov test is borderline significant (*p *= 0.018).

In the erbB2+ subgroup of the pooled cohort the Kolmogorov-Smirnov test significantly rejects the null hypothesis for various measures (see Additional file 4: Supplemental Figure 4). Some of the prognostic genes are at the top of the ranked gene lists based on VRS, WVRS, increasing kurtosis, the bimodality index and the likelihood ratio. The smallest p-value can be observed for the negative kurtosis (*p *< 10^-9^). Here, for the outlier-sum statistic no significant result is obtained (*p *= 0.625). In the ESR1-/erbB2- subgroup the only considerable enrichment can be observed for negative kurtosis (*p *< 10^-4^, see Additional file 5: Supplemental Figure 5).

It is important to note that the pooling of data sets can be problematical since in some cases bimodal expression distributions are an artifact of the pooling of experiments (see Additional file 6: Supplemental Figure 6).

### Analysis of established genes with bimodal distribution

**Figure 9:**
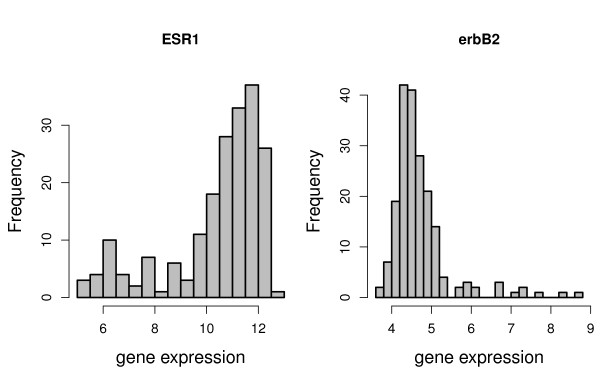
**Histograms of ESR1 and erbB2 expression values**.

Techniques for the identification of bimodally expressed genes should be able to identify genes, whose bimodal distribution is already known. The best established genes with bimodal frequency distributions in breast cancer are the estrogen receptor (ESR1) and erbB2 [1,2]. Histograms of the expression profiles of these genes in our data set are shown in Figure 9 and clearly indicate subgroups.

The ranks of these two genes based on the different bimodality scores are presented in Table 5. For many scores, the genes obtain small ranks. ESR1 is ranked by the bimodality index and VRS at position 50 and 92, respectively, and also has small ranks for WVRS, the outlier-sum statistic, and the likelihood ratio. Only for the dip statistic and for the kurtosis ESR1 obtains medium ranks.

**Table 5:** Ranks of known bimodal genes

score	ESR1	erbB2
VRS	92	808
WVRS	204	21505
dip	9055	15987
-(kurtosis)	11595	1023
likelihood ratio	623	426
bimodality index	50	338
outlier sum	296	480

The ranks of the known bimodal genes erbB2 and ESR1 based on the seven scores.

ErbB2 also has the smallest rank when using the bimodality index (338) and has small ranks for VRS, the outlier-sum statistic, and the likelihood ratio. The only extreme difference can be observed for WVRS, where erbB2 has rank 21505. The large value of WVRS is due to the large variance in the smaller group. Dip finds groups with almost equal sizes and thus fails to identify the groups with unequal sizes generated by ESR1 and erbB2 in our data set.

## Discussion and Conclusion

Genome-wide identification of genes with bimodal expression distributions is relevant since distinguishability of high and low expression groups is easier compared to genes with unimodal gene expression distributions. In addition, such methods may be helpful for the identification of candidate genes that are targets of somatic or germ-line mutations.

In the present study, we compared seven scores for the genome-wide identification of bimodal genes. Interestingly, the expression distributions of genes identified by the different scores showed characteristic differences. In general, two different concepts can be distinguished, namely the splitting of observations into two separated groups and the identification of a mixture of a reference group and outlier observations. The splitting approaches are the following: The dip test is a nonparametric test of unimodality. In our context, it identifies genes with approximately similar sizes of low and high expression groups. Genes with negative kurtosis values also show bimodal densities with almost equal mass of the groups or with flat distributions. The top genes according to the bimodality index mostly have bimodal distributions with unequal group sizes.

The outlier approaches are the correlated measures log-likelihood ratio, WVRS, kurtosis, and the outlier-sum statistic. Genes with large positive kurtosis have densities with a large group and a small group containing only a few patients. The likelihood ratio and the outlier-sum statistic both find genes with heavy tails in their expression profiles. In our evaluation, usually the smaller group showed higher expression values.

The clustering-based approach VRS seems to be a compromise, see Figure 3(a). Both genes identified by the splitting methods and by the outlier methods are ranked top. VRS is also suitable for detecting splits with corresponding noticeable different group sizes.

The second result of our analysis is which measures are best suited for finding prognostic genes. Genome-wide analysis of all genes in the Mainz cohort study demonstrates that the prognostic genes are clearly overrepresented among the genes identified by various measures. The smallest p-values of the Kolmogorov-Smirnov test that evaluates the entire bimodality ranking at once were obtained for the likelihood ratio, the outlier-sum statistic and the bimodality index. The outlier-sum approach produces a gene list in which prognostic genes have small ranks (*p *< 10^-12^). There is a strong Pearson correlation between the log-likelihood ratio and the outlier-sum statistic (0.863) and the p-value of the Kolmogorov-Smirnov test for the likelihood ratio is also very small (*p *< 10^-12^). Both methods identify similar types of distributions. Many of the prognostic genes have densities with heavy tails. The bimodality index finds genes with bimodal densities where the group sizes can be unequal. The prognostic genes are clearly overrepresented among the top-scoring genes according to the bimodality index (*p *< 10^-12^). The next best measure is VRS (Table 4). This means that both splitting methods (like the bimodality index) and outlier methods (like the likelihood ratio) can be useful for finding interesting prognostic genes. For the weighted cluster score WVRS, the overall overrepresentation of the prognostic genes among the top-scoring genes is not significant (*p *= 0.547). However, many of the genes with large WVRS values obtain very small p-values according to the logrank test. The expression profiles of these genes show a unimodal distribution and a smaller group with large variance. The validation of our results using the Rotterdam cohort showed that the prognostic genes were also clearly overrepresented among the top-genes according to the likelihood ratio and the outlier-sum statistic.

Another insight is that, in concordance with previous findings, only small groups of patients who strongly overexpress certain prognostic factors show worse prognosis, whereas dichotomization of the same factors at the median results in no or a worse prognostic power [1]. Often, RNA expression levels in the high expression groups are so high above those of the low expression groups that the overlap is virtually zero. Therefore, outlier scores like the kurtosis may be particularly valuable for screening for genes to be used subsequently in prognostic signatures. For this type of genes, often, the number of patients in the high expression group is very small. However, combination of several such bimodal genes may nevertheless allow the identification of a high risk group of an adequate size.

The dip is certainly not a technique of first choice for preselecting genes for prognostic signatures. However, dip may be ideal for the identification of genes whose bimodal expression may be caused by genetic polymorphisms with frequent alleles which is of interest in drug metabolism and other fields of toxicological research [33-35].

Among the identified genes several have not yet been recognized to be prognostic and bimodally expressed in breast cancer. An extensive overview over the top identified genes in our study regarding their gene function and the role of polymorphisms in tumor development is presented in Additional file 7: Supplemental Table 1. Important examples are the genes MAGEA2 and MAGEA4 that play central roles in immune response. Both have not yet been reported to be prognostic in breast cancer nor to show bimodal distributions. Further examples are desmoglein 1 (DSG1) which is involved in intercellular junction of epithelial cells, the phase II metabolizing enzyme UGT1A@, the cadherin-binding protein CTNNA2, and myosin heavy chain 7 (MYH7), which so far has been associated with heart disease but not with cancer. Examples where the polymorphism or its prognostic relevance have already been reported are the transcription factor FOXA1 and HAPLN1. FOXA1 has already been shown to be a predictor of good outcome in breast cancer, in agreement with the result of the present study. HAPLN1 conveys tumorigenic properties when overexpressed in mesothelioma cells, but SNPs of the HAPLN1 locus were not found to be associated with response to interferon beta and a prognostic role in breast cancer has not been reported yet (for details and references see Additional file 7).

In summary, we have established efficient techniques for the genome-wide identification of genes with bimodal expression distributions and we have analyzed their ability to generate patient splits with prognostic relevance. Future research includes the analysis of multivariate bimodality measures and the construction of prognostic signatures by combining several genes with bimodal expression distributions.

## Availability

R code for measures of bimodality and linking them to survival data is available at http://www.statistik.tu-dortmund.de/genetik-publikationen-bimodality.html.

## Authors' contributions

BH, JGH and JR developed the ideas for the manuscript, BH performed the statistical analyses, MS, MCG, WS and JGH generated and provided the data, JGH compiled the Supplemental Table, BH and JR drafted the manuscript, all authors read and approved the manuscript.

## Supplementary Material

Additional file 1 **Supplemental Figure 1**. Plots of the Kolmogorov-Smirnov type test for the 8 scores in the Mainz cohort using 250 prognostic genes. The genes are ordered according to the bimodality measures. We define a set of *N*_1 _prognostic genes by choosing the genes with the smallest p-values of the logrank test. We define *N*_0 _= *N *- *N*_1 _where *N *is the total number of genes. For the Kolmogorov-Smirnov type test a running-sum statistic is calculated by going through the ranked gene list. If a gene belongs to the set of prognostic genes *N*_0 _is added, if it does not belong to this set *N*_1 _is subtracted. The statistic is constructed such that the total sum is always 0. The maximal deviation from 0 is calculated which is large if there is an enrichment of the top-scoring genes with prognostic genes. On the x-axis of this plot the genes are ranked according to the particular score. On the y-axis is the running-sum statistic. The marks at the zero line indicate the positions of the prognostic genes in the ranked gene list. Click here for file

Additional file 2 **Supplemental Figure 2**. Plots of the Kolmogorov-Smirnov type test for the 8 scores in the Rotterdam cohort using 250 prognostic genes. Click here for file

Additional file 3 **Supplemental Figure 3**. Plots of the Kolmogorov-Smirnov type test for the 8 scores in the ESR1+/erbB2- subgroup of the pooled cohort using 250 prognostic genes. In the ESR1+/erbB2- subgroup of the pooled cohort the bimodality index has the smallest p-value of the logrank test (*p *< 10^-14^). The prognostic genes are clearly overrepresented among the first 9000 genes. A significant enrichment of the top scoring genes with prognostic genes can also be observed for the likelihood ratio (*p *< 10^-8^) and for the outlier-sum statistic (*p *< 10^-5^). For the kurtosis score the prognostic genes have mainly negative values. There are also many genes with large positive kurtosis but these are not the top genes for positive kurtosis. For VRS the result of the Kolmogorov-Smirnov test is borderline significant (*p *= 0.018). For the other scores the null hypothesis of uniformly distributed ranks of the prognostic genes can not be rejected. Click here for file

Additional file 4 **Supplemental Figure 4**. Plots of the Kolmogorov-Smirnov type test for the 8 scores in the erbB2+ subgroup of the pooled cohort using 250 prognostic genes. In the erbB2+ subgroup of the pooled cohort the Kolmogorov-Smirnov test significantly rejects the null hypothesis for various measures. In the corresponding plots one can see that some of the prognostic genes are at the top of the ranked gene lists based on VRS, WVRS, increasing kurtosis, the bimodality index and the likelihood ratio. The smallest p-value can be observed for the negative kurtosis (*p *< 10^-9^). The prognostic genes mostly have expression distributions with two major subgroups. Here, for the outlier-sum statistic no significant result is obtained (*p *= 0.625). Click here for file

Additional file 5 **Supplemental Figure 5**. Plots of the Kolmogorov-Smirnov type test for the 8 scores in the ESR1-/erbB2- subgroup of the pooled cohort using 250 prognostic genes. In the ESR1-/erbB2- subgroup of the pooled cohort the only considerable enrichment can be observed for negative kurtosis (*p *< 10^-4^). For WVRS the p-value is also smaller than 5% (*p *= 0.025), but in the corresponding plot no overrepresentation of prognostic genes among the top-scoring genes is visible. Click here for file

Additional file 6 **Supplemental Figure 6**. Scatterplots of the expression values of the 5 top genes according to negative kurtosis in the ESR1+/erbB2- subgroup of the pooled cohort. The colors indicate the two groups obtained by model-based clustering, the vertical lines separate the 4 data sets. This plot shows that in some cases bimodal expression distributions are an artifact of the pooling of experiments. For example, for the second gene (VAMP3) the first and third data set contain only high expression values, the second almost only low values. Therefore, pooling is dangerous. However, for some cases the different data sets yield consistent results, see, e.g., the genes TFAP2B and HLA-DQA1. Click here for file

Additional file 7 **Supplemental Table 1**. Overview over function and polymorphisms of the genes with bimodal expression distribution. Special care was given to identify publications reporting about polymorphisms of the genes of interest in tumor tissue or a possible role in tumor development. For most of the genes identified as bimodal in the present study no evidence for functional polymorphisms in tumor tissue has been published. Click here for file
