# Supplementary figures and images for: Comparison of scores for bimodality of gene expression distributions and genome-wide evaluation of the prognostic relevance of high-scoring genes
Source: BMC Bioinformatics. 2010 May 25;11:276. doi: 10.1186/1471-2105-11-276 (PMC2892466; doi:10.1186/1471-2105-11-276)

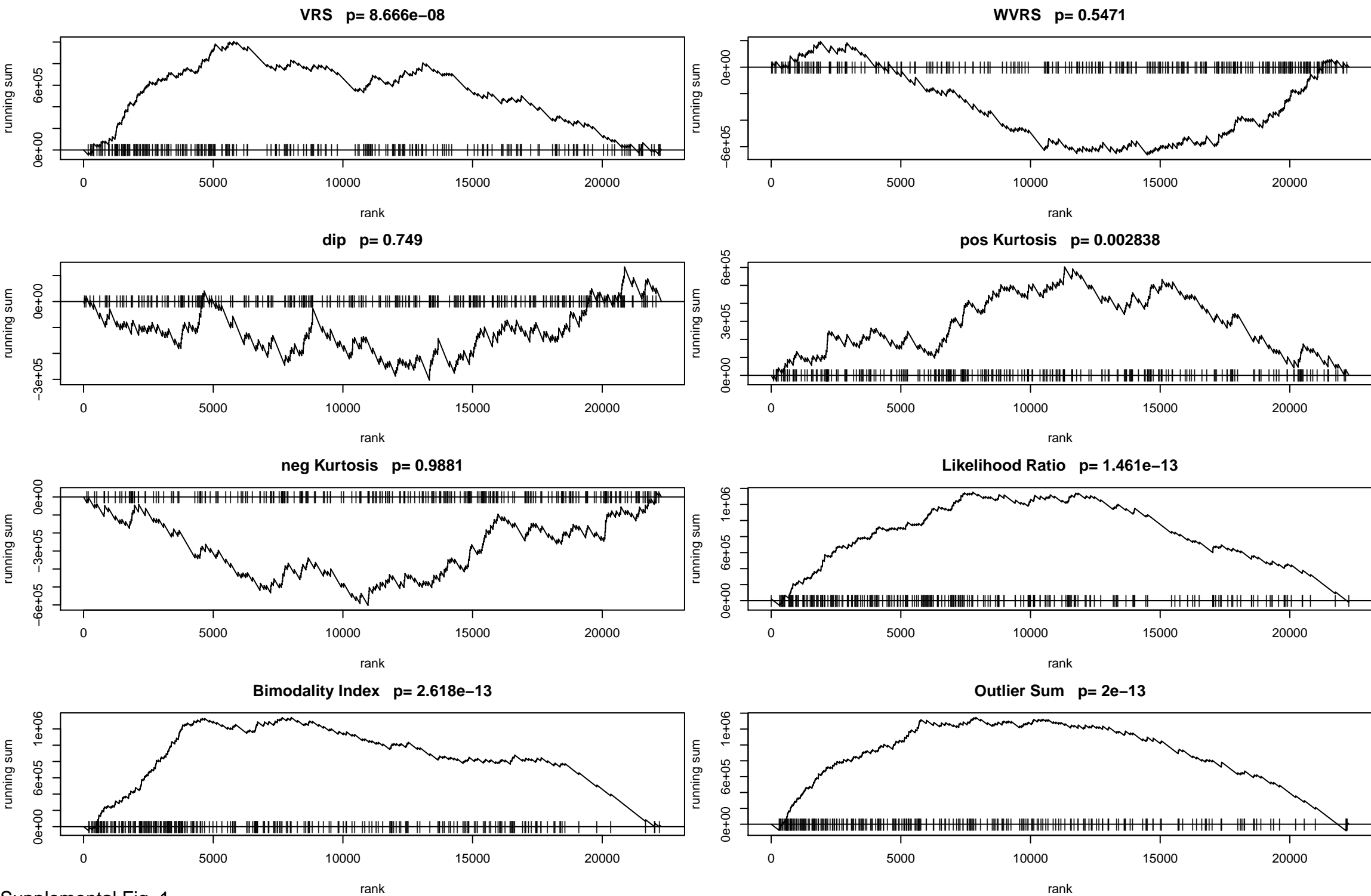

Supplemental Fig. 1

Supplement: Additional file 1 — Supplemental Figure 1. Plots of the Kolmogorov-Smirnov type test for the 8 scores in the Mainz cohort using 250 prognostic genes. The genes are ordered according to the bimodality measures. We define a set of N1 prognostic genes by choosing the genes with the smallest p-values of the logrank test. We define N0 = N - N1 where N is the total number of genes. For the Kolmogorov-Smirnov type test a running-sum statistic is calculated by going through the ranked gene list. If a gene belongs to the set of prognostic genes N0 is added, if it does not belong to this set N1 is subtracted. The statistic is constructed such that the total sum is always 0. The maximal deviation from 0 is calculated which is large if there is an enrichment of the top-scoring genes with prognostic genes. On the x-axis of this plot the genes are ranked according to the particular score. On the y-axis is the running-sum statistic. The marks at the zero line indicate the positions of the prognostic genes in the ranked gene list. [file 1471-2105-11-276-S1.PDF]

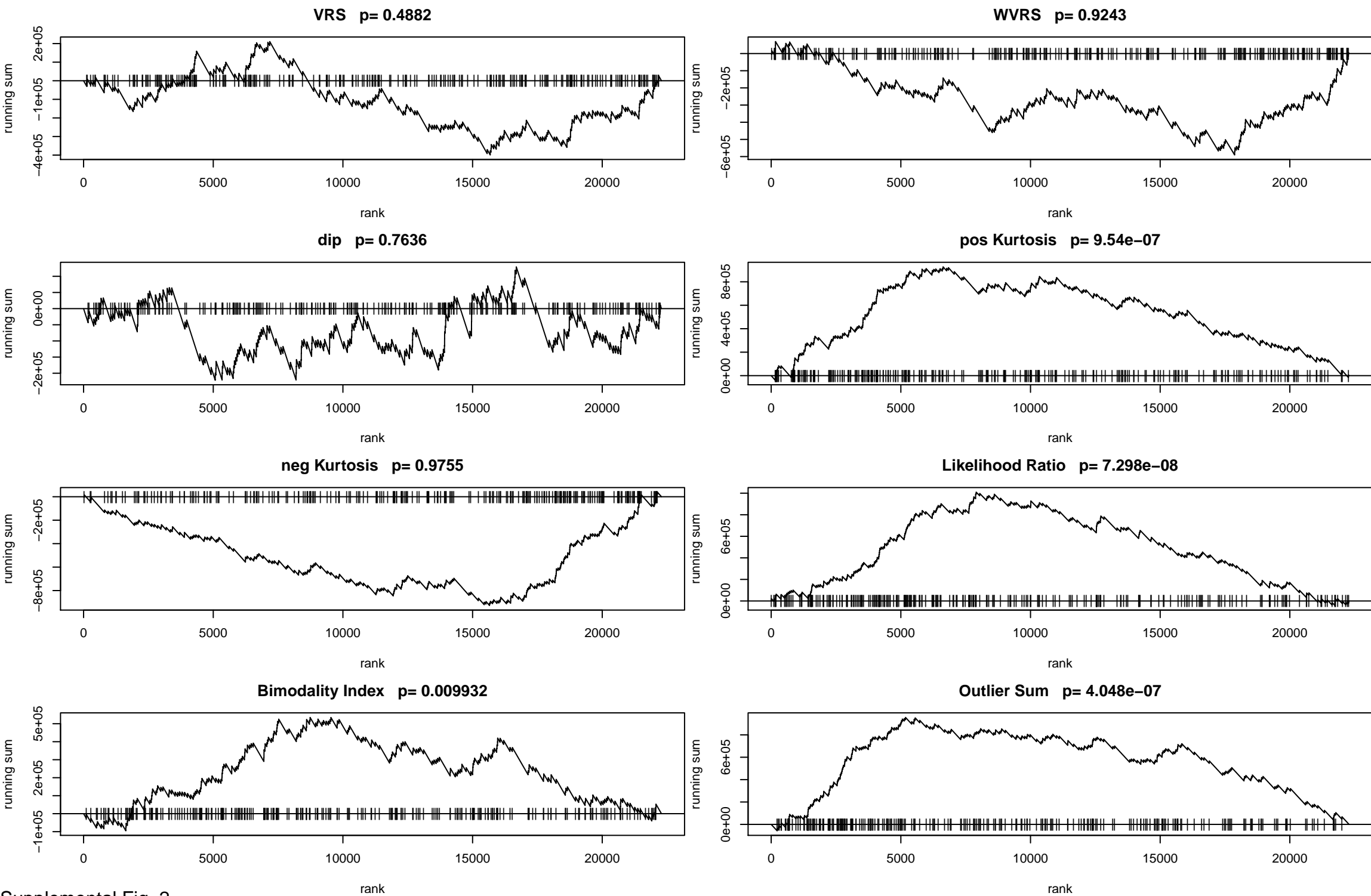

Supplemental Fig. 2

Supplement: Additional file 2 — Supplemental Figure 2. Plots of the Kolmogorov-Smirnov type test for the 8 scores in the Rotterdam cohort using 250 prognostic genes. [file 1471-2105-11-276-S2.PDF]

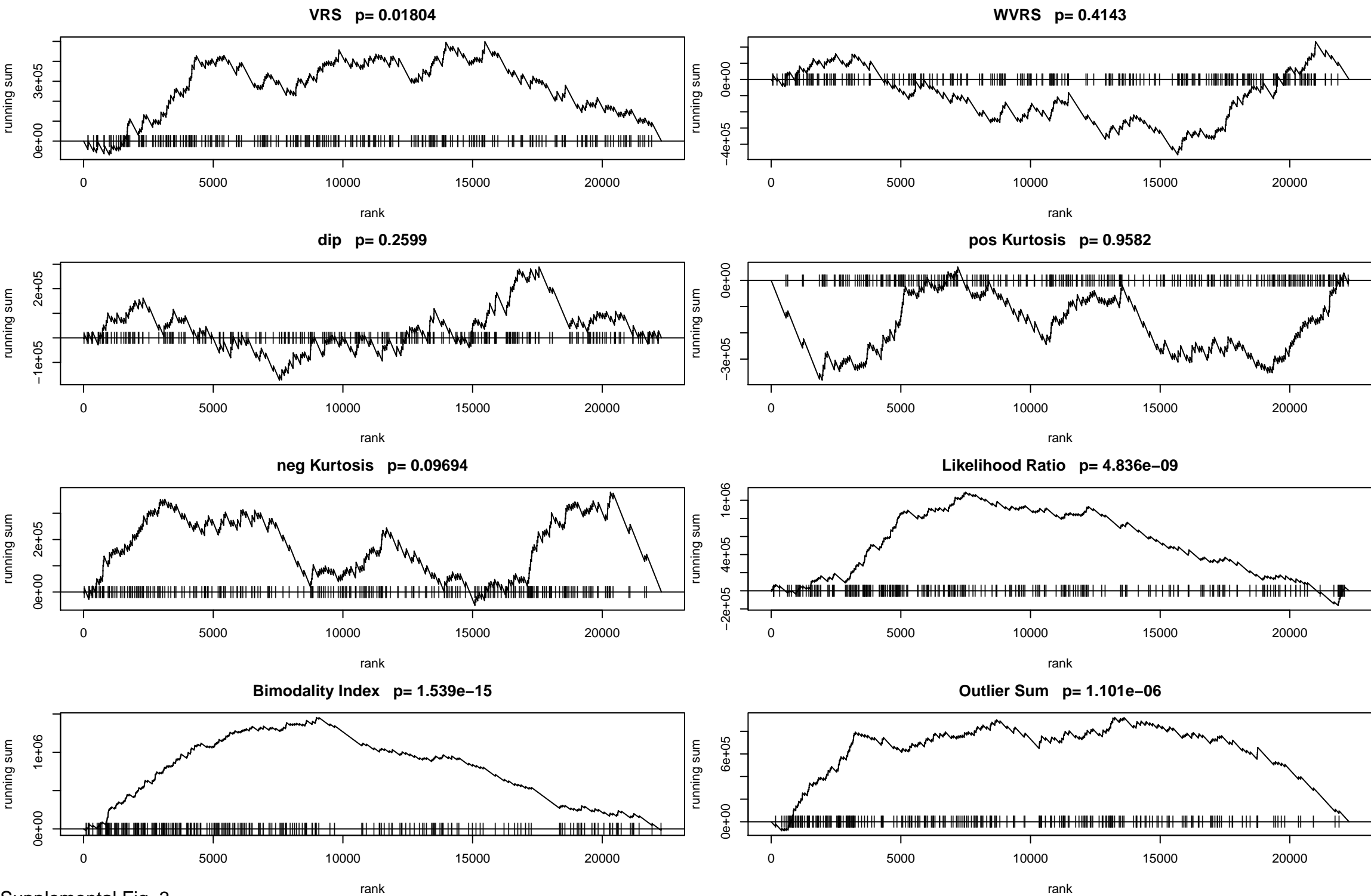

Supplemental Fig. 3

Supplement: Additional file 3 — Supplemental Figure 3. Plots of the Kolmogorov-Smirnov type test for the 8 scores in the ESR1+/erbB2- subgroup of the pooled cohort using 250 prognostic genes. In the ESR1+/erbB2- subgroup of the pooled cohort the bimodality index has the smallest p-value of the logrank test (p < 10-14). The prognostic genes are clearly overrepresented among the first 9000 genes. A significant enrichment of the top scoring genes with prognostic genes can also be observed for the likelihood ratio (p < 10-8) and for the outlier-sum statistic (p < 10-5). For the kurtosis score the prognostic genes have mainly negative values. There are also many genes with large positive kurtosis but these are not the top genes for positive kurtosis. For VRS the result of the Kolmogorov-Smirnov test is borderline significant (p = 0.018). For the other scores the null hypothesis of uniformly distributed ranks of the prognostic genes can not be rejected. [file 1471-2105-11-276-S3.PDF]

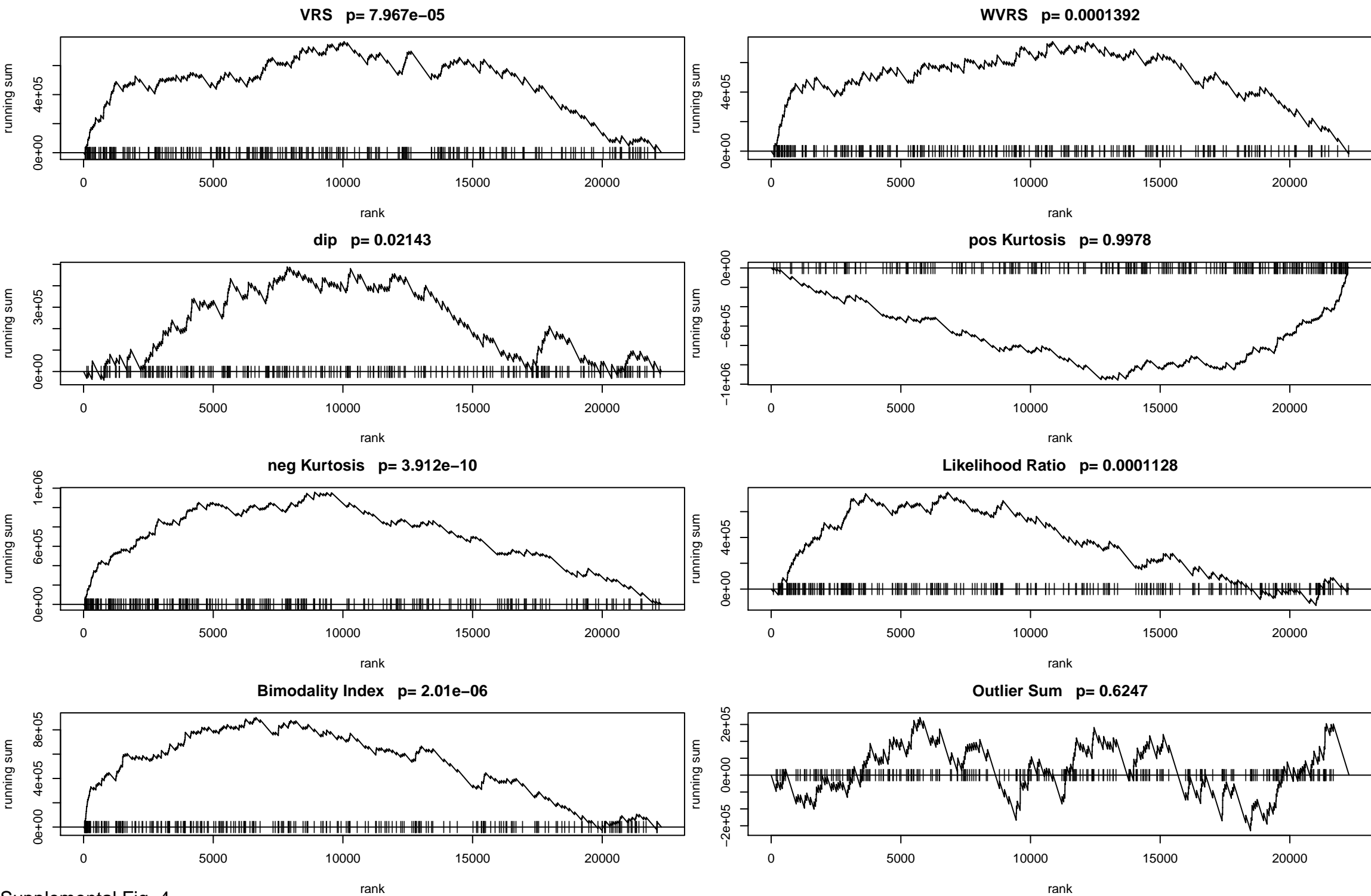

Supplemental Fig. 4

Supplement: Additional file 4 — Supplemental Figure 4. Plots of the Kolmogorov-Smirnov type test for the 8 scores in the erbB2+ subgroup of the pooled cohort using 250 prognostic genes. In the erbB2+ subgroup of the pooled cohort the Kolmogorov-Smirnov test significantly rejects the null hypothesis for various measures. In the corresponding plots one can see that some of the prognostic genes are at the top of the ranked gene lists based on VRS, WVRS, increasing kurtosis, the bimodality index and the likelihood ratio. The smallest p-value can be observed for the negative kurtosis (p < 10-9). The prognostic genes mostly have expression distributions with two major subgroups. Here, for the outlier-sum statistic no significant result is obtained (p = 0.625). [file 1471-2105-11-276-S4.PDF]

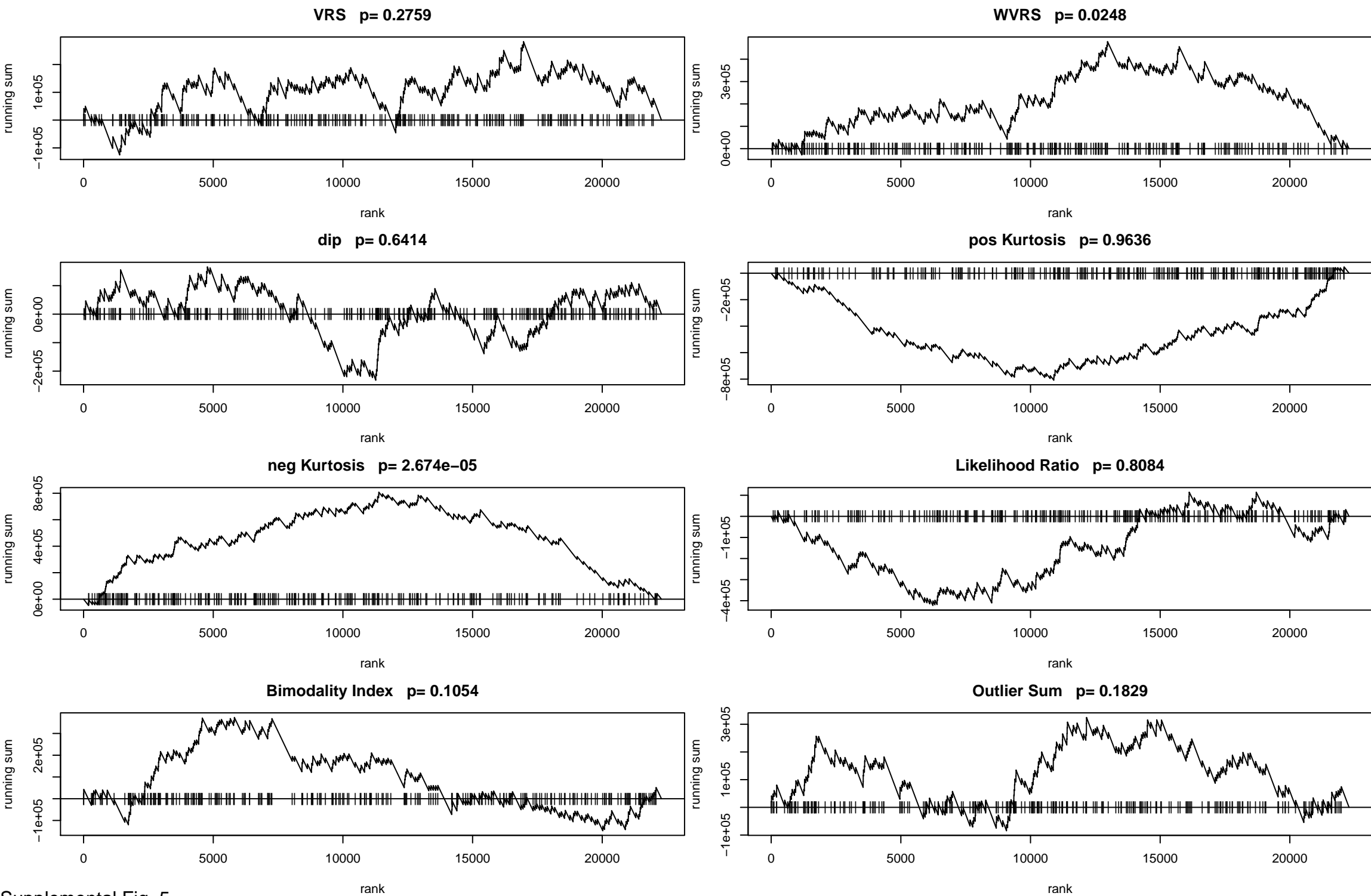

Supplemental Fig. 5

Supplement: Additional file 5 — Supplemental Figure 5. Plots of the Kolmogorov-Smirnov type test for the 8 scores in the ESR1-/erbB2- subgroup of the pooled cohort using 250 prognostic genes. In the ESR1-/erbB2- subgroup of the pooled cohort the only considerable enrichment can be observed for negative kurtosis (p < 10-4). For WVRS the p-value is also smaller than 5% (p = 0.025), but in the corresponding plot no overrepresentation of prognostic genes among the top-scoring genes is visible. [file 1471-2105-11-276-S5.PDF]

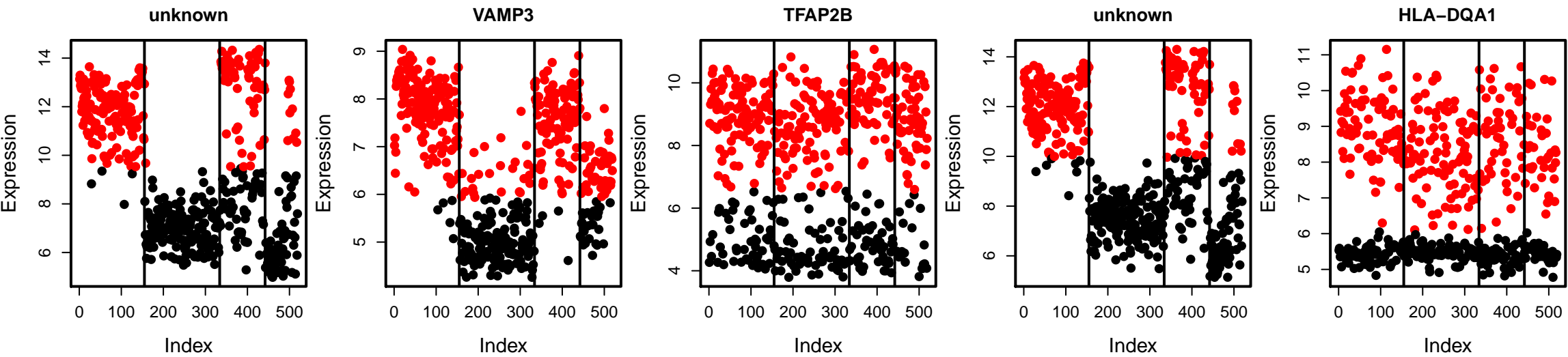

Supplemental Fig. 6

Supplement: Additional file 6 — Supplemental Figure 6. Scatterplots of the expression values of the 5 top genes according to negative kurtosis in the ESR1+/erbB2- subgroup of the pooled cohort. The colors indicate the two groups obtained by model-based clustering, the vertical lines separate the 4 data sets. This plot shows that in some cases bimodal expression distributions are an artifact of the pooling of experiments. For example, for the second gene (VAMP3) the first and third data set contain only high expression values, the second almost only low values. Therefore, pooling is dangerous. However, for some cases the different data sets yield consistent results, see, e.g., the genes TFAP2B and HLA-DQA1. [file 1471-2105-11-276-S6.PDF]
