# Supplementary material for: Comparison of scores for bimodality of gene expression distributions and genome-wide evaluation of the prognostic relevance of high-scoring genes
Source: BMC Bioinformatics. 2010 May 25;11:276. doi: 10.1186/1471-2105-11-276 (PMC2892466; doi:10.1186/1471-2105-11-276)
Supplement: Additional file 7 — Supplemental Table 1. Overview over function and polymorphisms of the genes with bimodal expression distribution. Special care was given to identify publications reporting about polymorphisms of the genes of interest in tumor tissue or a possible role in tumor development. For most of the genes identified as bimodal in the present study no evidence for functional polymorphisms in tumor tissue has been published. [file 1471-2105-11-276-S7.PDF]

Supplemental Table 1: Overview over function and polymorphisms of the genes with bimodal expression distribution. Special care was given to identify publications reporting about polymorphisms of the genes of interest in tumor tissue or a possible role in tumor development. For most of the genes identified as bimodal in the present study no evidence for functional polymorphisms in tumor tissue has been published.

	**Gene function**	**Role of polymorphisms in tumor development**
ACE	ANGIOTENSIN I-CONVERTING ENZYME 2 ACE2 catalyzes the conversion of angiotensin I, plays a role in the renin-angiotensin system and mediates cardiovascular and renal functions.	Decreased expression of ACE2 is associated with progression of pancreatic ductal adenocarcinoma (Zhou *et al.*, 2009). ACE2 polymorphisms relevant for tumor development have not been reported.
ALB	ALBUMIN comprises about one-half of the blood serum protein.	Not reported
ASCL1	ACHAETE-SCUTE COMPLEX, DROSOPHILA, HOMOLOG OF, 1 is a basic helix-loop-helix transcription factor.	ASCL1 has been reported to be a marker for small cell lung carcinomas with neuroendocrine features (Westerman *et al.*, 2002).
CALCA	CALCITONIN/CALCITONIN-RELATED POLYPEPTIDE, ALPHA (CALCITONIN) is synthesized by the parafollicular cells of the thyroid. It causes reduction in serum calcium, an effect opposite to that of parathyroid hormone (PTH) (Costante *et al.*, 2007). An inverse association of dietary calcium intake with the risk of ovarian cancer has been reported (Goodman *et al.*, 2005).	An association of a CALCA gene polymorphism and ovarian cancer risk has been reported (Goodman *et al.*, 2005).
CARTPT	COCAINE- AND AMPHETAMINE-REGULATED TRANSCRIPT PROTEIN PRECURSOR (CART) plays a role in reward, feeding, and stress, and functions as an endogenous psychostimulant.	Not reported
CHGB	CHROMOGRANIN B is a secretory granule matrix protein expressed in a wide variety of endocrine cells and neurons (Natori et al., 1998).	CHGB may play a role in an early phase of breast cancer development (Kimura *et al.*, 2002). CHGB variants may be involved in the susceptibility to schizophrenia (Iijima *et al.*, 2004) but polymorphisms relevant for tumor development have not been reported.
CRISP3	CYSTEINE-RICH SECRETORY PROTEIN 3 is strongly expressed in neutrophils and in exocrine glands. A role in innate immune defense has been hypothesized. It is expressed at low levels in benign prostate tissues but highly overexpressed in prostate cancer. CRISP-3 has been reported to represent an independent predictor of recurrence after radical prostatectomy (Bjartell *et al.*, 2007).	A polymorphism within the CRISP3 gene is associated with stallion fertility in Hanoverian warmblood horses (Hamann *et al.*, 2007) but no role in tumor development has been described.

	Gene function	Role of polymorphisms in tumor development
CTNNA2	CATENIN, ALPHA-2 is a cadherin-binding protein, widely expressed in the nervous system, which plays a role in cadherin-mediated cell-cell adhesion (Uemura and Takeichi, 2006). A role in tumor development has not been reported.	Not reported.
DSCC1	DEFECTIVE IN SISTER CHROMATID COHESION PROTEIN 1 HOMOLOG (also: DCC1) is involved in DNA damage response.	DSCC1 is located at frequently amplified hotspots on chromosome 8q22.3 in breast cancer (Chin *et al.*, 2007).
DSG1	DESMOGLEIN 1 is involved in intercellular junction of epithelial cells. Loss of DSG1 may be associated with worse prognosis of carcinomas (Wong *et al.*, 2008).	A possible role of DSG1 SNPs in the susceptibility to exudative epidermitis has been discussed (Daugaard *et al.*, 2007). A role in tumor development has not been described.
ERAP2	LEUKOCYTE-DERIVED ARGININE AMINOPEPTIDASE is required for peptide trimming in order to generate HLA class I-binding peptides. Imbalanced expression of ERAP2 may cause improper antigen processing and favor tumor escape from the immune surveillance (Qu *et al.*, 2007).	A functional polymorphism of the ERAP2 gene was not associated with type 1 diabetes (Qu *et al.*, 2007). An association of ERAP2 with tumor development has not been reported.
FABP7	FATTY ACID-BINDING PROTEIN 7 is a cytosolic protein that enhances intracellular transfer of fatty acids.	FABP7 is associated with the basal phenotype and patient outcome in human breast cancer (Zhang *et al.*, 2009).
FAM5C	FAMILY WITH SEQUENCE SIMILARITY 5, MEMBER C is localized to the mitochondria. Over-expression may lead to increased proliferation, migration, and invasion.	FAM5C is over-expressed in human pituitary tumors but expression is decreased in tongue squamous cell carcinoma compared to normal tissue (Kuroiwa *et al.*, 2009).
FGA	FIBRINOGEN, A ALPHA POLYPEPTIDE, Fibrinogen is a plasma glycoprotein involved in coagulation, synthesized in the liver, composed of subunits alpha (FGA), beta (FGB), and gamma (FGG).	Not reported
FGB	FIBRINOGEN, B BETA POLYPEPTIDE Fibrinogen is a plasma glycoprotein involved in coagulation, synthesized in the liver, composed of subunits alpha (FGA), beta (FGB), and gamma (FGG).	Not reported
FOXA1	FORKHEAD BOX A1 (HEPATOCYTE NUCLEAR FACTOR 3-ALPHA) is a transcriptional activator originally described for liver-specific transcripts such as albumin and transthyretin that also play a role in several carcinomas. For example, FOXA1 was reported to be a predictor of good outcome in breast cancer (Albergaria *et al.*, 2009) and represents a potential oncogene in anaplastic thyroid carcinoma (Nucera *et al.*, 2009).	Not reported

	Gene function	Role of polymorphisms in tumor development
GLUD2	GLUTAMATE DEHYDROGENASE 2 catalyzes the reversible oxidative deamination of glutamate to alpha-ketoglutarate. Deficiency has been reported in patients with neurodegenerative disorders.	Not reported
GRIA2	GLUTAMATE RECEPTOR, IONOTROPIC is a ligand-activated cation channel that mediates the fast component of excitatory postsynaptic currents in neurons.	GRIA2 has been described as a marker genes for neuroendocrine carcinoma cells (Leja *et al.*, 2009). A role of polymorphisms in cancer progression has not been reported.
HAPLN1	HYALURONAN AND PROTEOGLYCAN LINK PROTEIN 1 increased tumorigenic properties when overexpressed in mesothelioma cells (Ivanova *et al.*, 2009).	Three SNPs in the HAPLN1 locus were not associated with treatment response to interferon-beta (Cénit *et al.*, 2009). An association of HAPLN1 polymorphisms with tumor development has not been reported.
HLA-DQA1	MAJOR HISTOCOMPATIBILITY COMPLEX, CLASS II, DQ alpha 1 plays a role in the immune system by presenting extracellular protein derived peptides. Class II molecules are expressed in lymphocytes, dendritic cells or macrophages.	The alpha chain and the beta chain of HLA-DQA1 contain polymorphisms relevant for peptide binding, resulting in up to 4 different molecules. The role of these polymorphisms has been studied in several types of carcinomas. Examples are:<br><br>HLA-DQA1 and -DQB1 locus comparison of allele frequencies between breast cancer patients and healthy controls showed no significant difference (Chen *et al.*, 2007).<br><br>Male childhood common acute lymphoblastic leukaemia patients had a higher frequency of DQA1*0101/*0104 and DQB1*0501 than appropriate control subjects (Taylor *et al.*, 1998)).<br><br>The DQA1*0102-DQB1*0602 haplotype is overrepresented among HPV-positive cases compared with controls (Helland *et al.*, 1998)
HLA-DQB1	MAJOR HISTOCOMPATIBILITY COMPLEX, CLASS II, DQ BETA-1 plays a role in the immune system. It presents peptides derived from extracellular proteins.	Variation in HLA-DQB1have been reported to influence the risk of several carcinomas including glioma (Torre *et al.*, 2009), cervical cancer (Liang *et al.*, 2008; Madeleine *et al.*, 2008) and breast cancer (Baccar Harrath *et al.*, 2006).
HLA-DRB4	MAJOR HISTOCOMPATIBILITY COMPLEX, CLASS II, DR BETA-3 is a class II major histocompatibility complex molecule that presents antigens to CD4-positive cells.	An association of HLA-DRB4 polymorphisms with chronic lymphocytic leukemia (Mueller and Machulla, 2002), childhood ALL (Dorak *et al.*, 2002) has been reported.
HTR2C	5-@HYDROXYTRYPTAMINE RECEPTOR 2C binds to distinct cell surface receptor subtypes in the mammalian nervous system.	Not reported

	Gene function	Role of polymorphisms in tumor development
IQGAP1	IQ MOTIF-CONTAINING GTPase-ACTIVATING PROTEIN 1 is a scaffold protein that binds to a wide variety of targets and modulates several cellular activities, including cell-cell adhesion, transcription, cytoskeletal architecture, and signaling pathways (Ren *et al.*, 2007). Proteins binding IQGAP1 include members of the MAP-kinase pathway, Cdc42, E-cadherin, Rac1, beta-catenin, and calmodulin. Evidence suggests that IQGAP1 is an oncogene (White *et al.*, 2009).	Not described
MAGEA2	MELANOMA ANTIGEN, FAMILY A2, directs the expression of tumor antigens that are recognized by autologous cytolytic T lymphocytes. MAGE-A genes are normally expressed in the human germ line and overexpressed in various tumor types. They target p53 transactivation function through histone deacetylase recruitment and confer resistance to chemotherapeutic agents (Monte *et al.*, 2006).	Not reported
MAGEA3	MELANOMA ANTIGEN, FAMILY A, 3 MELANOMA ANTIGEN, FAMILY A6, directs the expression of tumor antigens that are recognized by autologous cytolytic T lymphocytes. MAGE-A genes are normally expressed in the human germ line and overexpressed in various tumor types.	Not reported
MAGEA4	MELANOMA ANTIGEN, FAMILY A4, belongs to the same family as MAGEA2 described above. MAGE-A4 is expressed in several carcinomas and may sensitize malignancies to apoptotic stimuli (Peikert *et al.*, 2006).	Not reported
MAGEA6	MELANOMA ANTIGEN, FAMILY A6, directs the expression of tumor antigens that are recognized by autologous cytolytic T lymphocytes. MAGE-A genes are normally expressed in the human germ line and overexpressed in various tumor types.	Not reported
MSLN	MESOTHELIN is a cell surface antigen with unknown function that is expressed by several types of human carcinomas.	A single nucleotide polymorphisms in the 3'untranslated region (3'UTR) of the mesothelin-encoded gene can affect mesothelin expression (Cristaudo *et al.*, 2009). A role of MSLN polymorphisms in tumor development and progression has not been reported.
MYH7	MYOSIN, HEAVY CHAIN 7 is involved in development of familial hypertrophic cardiomyopathy (Bashyam *et al.*, 2007).	A polymorphism of the MYH7 gene has been reported to be associated with familial hypertrophic cardiomyopathy (Bashyam *et al.*, 2007). An association of MYH7 with tumor development has not been published.

	Gene function	Role of polymorphisms in tumor development
NLRP2	NLR FAMILY, PYRIN DOMAIN-CONTAINING 2 plays a role in establishing genomic imprinting in humans (Meyer *et al.*, 2009). NLRP2 also inhibits the NF-kappaB signaling pathway modulating the inflammatory response (Fontalba *et al.*, 2007).	A germline mutation in NLRP2 is associated with the familial imprinting disorder Beckwith-Wiedemann Syndrome (Meyer *et al.*, 2009). A polymorphism of NLRP2 may contribute to the amplification of inflammatory responses (Fontalba *et al.*, 2007).
PCSK1	PROPROTEIN CONVERTASE, SUBTILISIN/KEXIN-TYPE 1 processes proinsulin and proglucagon (Jansen *et al.*, 1995), is upregulated in soft-part sarcoma (Stockwin *et al.*, 2009) and alters breast cancer cell growth in response to estrogen and tamoxifen (Stockwin *et al.*, 2009).	Variants of PCSK1 confer risk of obesity (Benzinou *et al.*, 2008). PCSK1 is located in the centromeric region of rat chromosome 2 (2q1) that harbors unidentified quantitative trait loci of genes that control tumor growth or development (Quan *et al.*, 2000).
PEG10	PATERNALLY EXPRESSED GENE 10 interacts with the cytoplasmic domain of ALK1, a TGF-beta receptor (Lux *et al.*, 2005).	PEG10 is regulated by E2Fs and may play a role in carcinogenesis, such as development of hepatocellular cancer (Wang *et al.*, 2008). Polymorphisms relevant for tumor development have not been reported.
PSD3	PH AND SEC7 DOMAIN-CONTAINING PROTEIN 3	PSD3 was reported as a candidate gene involved in breast cancer metastasis (Thomassen *et al.*, 2009). Polymorphisms relevant for tumor development have not been reported.
PSPHL	PHOSPHOSERINE PHOSPHATASE-LIKE is upregulated in Fanconi anemia but downregulated or absent in normal fibroblasts. It represents a homologue to the L-3-phosphoserine phosphatase (Planitzer *et al.*, 1998).	Not reported
S100A7	S100 CALCIUM-BINDING PROTEIN A7 is a chemotactic inflammatory protein for CD4+ T lymphocytes and neutrophils.	S100A7 is considered as a possible therapeutic target for inflammation and cancer (León *et al.*, 2009). Polymorphisms of S100A7 have been reported to be associated with allergic rhinitis (Bryborn *et al.*, 2008). However, an association between S100A7 variants and cancer progression has not yet been published.
SAT1	SPERMIDINE/SPERMINE N(1)-ACETYLTRANSFERASE 1 is a rate-limiting enzyme in the catabolic pathway of polyamine metabolism (Casero *et al.*, 1991). Some polyamine analogs can mimic induction of SAT1 and can cause a loss of polyamines. This may have utility in cancer chemotherapy (Pegg, 2008).	SAT1 Promoter polymorphisms have been reported to be associated with decreased SAT1 expression in suicide completers (Fiori *et al.*, 2009) and in anxiety (Vaquero-Lorenzo *et al.*, 2009). A role of SAT1 polymorphisms in tumor development has not yet been described.

	Gene function	Role of polymorphisms in tumor development
SCG3	SECRETOGRANIN III plays a role in development of obesity. SCG3 forms secretory granules with orexin, melanin-concentrating hormone (MCH), neuropeptide Y (NPY), and POMC in the hypothalamus (Hotta *et al.*, 2009). SCG3 mRNA was reported to provide a sensitive prognostic biomarker for neuroendocrine lung cancer (Moss *et al.*, 2009).	Polymorphisms of the SCG3 gene have been reported to influence the risk of obesity through regulation of hypothalamic neuropeptide secretion (Tanabe *et al.*, 2007).
SCGB1D2	LIPOPHILIN B (SCGB1D2) is a member of the secretoglobin superfamily.	SCGB1D2 is known to be expressed in breast cancer (Zafrakas *et al.*, 2006). However, no polymorphisms relevant for tumor development have been reported.
SLC1A1	SOLUTE CARRIER FAMILY 1 is a high-affinity glutamate transporter that also transports aspartate.	SLC1A1 is expressed and amplified in some carcinomas, such as head and neck squamous cell carcinomas (Järvinen *et al.*, 2008). Haplotypes have been reported to be associated with obsessive-compulsive disorder (Wendland *et al.*, 2009) but not with tumor development.
ST18	SUPPRESSION OF TUMORIGENICITY 18 has been described as a zinc-finger transcription factor that may function as a breast cancer tumor suppressor gene (Jandrig *et al.*, 2004) and regulates proapoptotic and proinflammatory gene expression (Yang *et al.*, 2008).	Not reported
TDRD1	TUDOR DOMAIN CONTAINING 12, function not known	Not reported
TFAP2B	TRANSCRIPTION FACTOR AP2-BETA is associated with insulin resistance and adiposity (Nordquist *et al.*, 2009). TFAP2B was reported to be differentially expressed between lobular and ductal breast carcinomas (Korkola *et al.*, 2003).	Genetic variants of TFAP2B have been reported to be associated with type 2 diabetes mellitus (Maeda *et al.*, 2005).
UGT1A@	UDP-GLYCOSYLTRANSFERASE 1 FAMILY, POLYPEPTIDE A1; UGT1A1 is a phase 2 drug metabolizing enzyme.	UGT1A1 genetic variants have a predictive role on the outcome of metastatic colorectal cancer patients treated with chemotherapy (Cecchin *et al.*, 2009) and influence metabolism associated cancer susceptibility (Girard *et al.*, 2008).
UGT2B4	URIDINE DIPHOSPHATE GLYCOSYLTRANSFERASE 2 FAMILY, MEMBER B4 is a phase 2 drug metabolizing enzyme.	UGT2B4 contains several polymorphisms that may influence hormone and drug metabolism (Maruo *et al.*, 2005) and may be associated with circulating sex hormone concentrations (Sparks *et al.*, 2004).
UGT8	URIDINE DIPHOSPHATE GLYCOSYLTRANSFERASE 8 is the first enzyme involved in complex lipid biosynthesis in the myelinating oligodendrocyte.	UGT8 belongs to a six-gene signature predicting breast cancer lung metastasis (Culhane and Quackenbush, 2009).

	Gene function	Role of polymorphisms in tumor development
UTS2	UROTENSIN II (UTS2) is a vasoconstrictor peptide that is associated with cardiovascular disease and also plays a role in glucose metabolism and insulin resistance (Jiang *et al.*, 2008). A role in tumor development has not been reported.	Polymorphisms of the UTS2 gene have been reported to be associated with the development of Type 2 diabetes (Wenyi *et al.*, 2003) and hypertension (Yi *et al.*, 2006).
VGLL1	VESTIGIAL-LIKE 1 belongs to a conserved family of transcription factors.	Not reported
ZIC1	ZINC FINGER PROTEIN OF CEREBELLUM 1 is a tumor suppressor gene silenced through promoter hypermethylation in gastric cancer (Wang *et al.*, 2009), also deregulated in endometrial cancer (Wong *et al.*, 2007).	Genetic variants in ZIC1, ZIC2, and ZIC3 are not major risk factors for neural tube defects in humans (Klootwijk *et al.*, 2004). No role of polymorphisms in tumor development has been described.

References

Albergaria, A., Paredes, J., Sousa, B., Milanezi, F., Carneiro, V., Bastos, J., Costa, S., Vieira, D., Lopes, N., Lam, E. W., Lunet, N., and Schmitt, F. (2009). Expression of FOXA1 and GATA-3 in breast cancer: the prognostic significance in hormone receptor-negative tumours. *Breast Cancer Res*, **11**(3), R40.

Baccar Harrath, A., Yacoubi Loueslati, B., Troudi, W., Hmida, S., Sedkaoui, S., Dridi, A., Jridi, A., Ben Ayed, F., Ben Rhomdhane, K., and Ben Ammar Elgaaied, A. (2006). HLA class II polymorphism: protective or risk factors to breast cancer in Tunisia? *Pathol Oncol Res*, **12**(2), 79–81.

Bashyam, M. D., Savithri, G. R., Gopikrishna, M., and Narasimhan, C. (2007). A p.R870H mutation in the beta-cardiac myosin heavy chain 7 gene causes familial hypertrophic cardiomyopathy in several members of an Indian family. *Can J Cardiol*, **23**(10), 788–790.

Benzinou, M., Creemers, J. W. M., Choquet, H., Lobbens, S., Dina, C., Durand, E., Guerardel, A., Boutin, P., Jouret, B., Heude, B., Balkau, B., Tichet, J., Marre, M., Potoczna, N., Horber, F., Stunff, C. L., Czernichow, S., Sandbaek, A., Lauritzen, T., Borch-Johnsen, K., Andersen, G., Kiess, W., Körner, A., Kovacs, P., Jacobson, P., Carlsson, L. M. S., Walley, A. J., Jørgensen, T., Hansen, T., Pedersen, O., Meyre, D., and Froguel, P. (2008). Common nonsynonymous variants in PCSK1 confer risk of obesity. *Nat Genet*, **40**(8), 943–945.

Bjartell, A. S., Al-Ahmadie, H., Serio, A. M., Eastham, J. A., Eggener, S. E., Fine, S. W., Udby, L., Gerald, W. L., Vickers, A. J., Lilja, H., Reuter, V. E., and Scardino, P. T. (2007). Association of cysteine-rich secretory protein 3 and beta-microseminoprotein with outcome after radical prostatectomy. *Clin Cancer Res*, **13**(14), 4130–4138.

Bryborn, M., Halldén, C., Säll, T., Adner, M., and Cardell, L. O. (2008). Comprehensive evaluation of genetic variation in S100A7 suggests an association with the occurrence of allergic rhinitis. *Respir Res*, **9**, 29.

Casero, R. A., Celano, P., Ervin, S. J., Applegren, N. B., Wiest, L., and Pegg, A. E. (1991). Isolation and characterization of a cDNA clone that codes for human spermidine/spermine N1-acetyltransferase. *J Biol Chem*, **266**(2), 810–814.

Cecchin, E., Innocenti, F., D'Andrea, M., Corona, G., Mattia, E. D., Biason, P., Buonadonna, A., and Toffoli, G. (2009). Predictive role of the UGT1A1, UGT1A7, and UGT1A9 genetic variants and their haplotypes on the outcome of metastatic colorectal cancer patients treated with fluorouracil, leucovorin, and irinotecan. *J Clin Oncol*, **27**(15), 2457–2465.

Cénit, M. D. C., Blanco-Kelly, F., de las Heras, V., Bartolomé, M., de la Concha, E. G., Urcelay, E., Arroyo, R., and Martínez, A. (2009). Glypican 5 is an interferon-beta response gene: a replication study. *Mult Scler*, **15**(8), 913–917.

Chen, P.-C., Tsai, E.-M., Er, T.-K., Chang, S.-J., and Chen, B.-H. (2007). HLA-DQA1 and -DQB1 allele typing in southern Taiwanese women with breast cancer. *Clin Chem Lab Med*, **45**(5), 611–614.

Chin, S. F., Teschendorff, A. E., Marioni, J. C., Wang, Y., Barbosa-Morais, N. L., Thorne, N. P., Costa, J. L., Pinder, S. E., van de Wiel, M. A., Green, A. R., Ellis, I. O., Porter, P. L., Tavaré, S., Brenton, J. D., Ylstra, B., and Caldas, C. (2007). High-resolution aCGH and expression profiling identifies a novel genomic subtype of ER negative breast cancer. *Genome Biol*, **8**(10), R215.

Costante, G., Meringolo, D., Durante, C., Bianchi, D., Nocera, M., Tumino, S., Crocetti, U., Attard, M., Maranghi, M., Torlontano, M., and Filetti, S. (2007). Predictive value of serum calcitonin levels for preoperative diagnosis of medullary thyroid carcinoma in a cohort of 5817 consecutive patients with thyroid nodules. *J Clin Endocrinol Metab*, **92**(2), 450–455.

Cristaudo, A., Foddis, R., Bonotti, A., Simonini, S., Vivaldi, A., Guglielmi, G., Bruno, R., Landi, D., Gemignani, F., and Landi, S. (2009). Polymorphisms within the putative micro-RNA binding sites of mesothelin gene are associated with the serum levels of the mesothelin-related protein. *Occup Environ Med*.

Culhane, A. C. and Quackenbush, J. (2009). Confounding effects in "A six-gene signature predicting breast cancer lung metastasis". *Cancer Res*, **69**(18), 7480–7485.

Daugaard, L., Andresen, L. O., and Fredholm, M. (2007). Investigation of SNPs in the porcine desmoglein 1 gene. *BMC Vet Res*, **3**, 4.

Dorak, M. T., Oguz, F. S., Yalman, N., Diler, A. S., Kalayoglu, S., Anak, S., Sargin, D., and Carin, M. (2002). A male-specific increase in the HLA-DRB4 (DR53) frequency in high-risk and relapsed childhood ALL. *Leuk Res*, **26**(7), 651–656.

Fiori, L. M., Mechawar, N., and Turecki, G. (2009). Identification and characterization of spermidine/spermine N1-acetyltransferase promoter variants in suicide completers. *Biol Psychiatry*, **66**(5), 460–467.

Fontalba, A., Gutierrez, O., and Fernandez-Luna, J. L. (2007). NLRP2, an inhibitor of the NF-kappaB pathway, is transcriptionally activated by NF-kappaB and exhibits a nonfunctional allelic variant. *J Immunol*, **179**(12), 8519–8524.

Girard, H., Butler, L. M., Villeneuve, L., Millikan, R. C., Sinha, R., Sandler, R. S., and Guillemette, C. (2008). UGT1A1 and UGT1A9 functional variants, meat intake, and colon cancer, among Caucasians and African-Americans. *Mutat Res*, **644**(1-2), 56–63.

Goodman, M. T., Ferrell, R., McDuffie, K., Thompson, P. J., Wilkens, L. R., Bushley, A. W., Tung, K.-H., Carney, M. E., and Ness, R. B. (2005). Calcitonin gene polymorphism CALCA-624 (T/C) and ovarian cancer. *Environ Mol Mutagen*, **46**(1), 53–58.

Hamann, H., Jude, R., Sieme, H., Mertens, U., Töpfer-Petersen, E., Distl, O., and Leeb, T. (2007). A polymorphism within the equine CRISP3 gene is associated with stallion fertility in Hanoverian warmblood horses. *Anim Genet*, **38**(3), 259–264.

Helland, A., Olsen, A. O., Gjøen, K., Akselsen, H. E., Sauer, T., Magnus, P., Børresen-Dale, A. L., and Rønningen, K. S. (1998). An increased risk of cervical intra-epithelial neoplasia grade II-III among human papillomavirus positive patients with the HLA-DQA1*0102-DQB1*0602 haplotype: a population-based case-control study of Norwegian women. *Int J Cancer*, **76**(1), 19–24.

Hotta, K., Hosaka, M., Tanabe, A., and Takeuchi, T. (2009). Secretogranin II binds to secretogranin III and forms secretory granules with orexin, neuropeptide Y, and POMC. *J Endocrinol*, **202**(1), 111–121.

Iijima, Y., Inada, T., Ohtsuki, T., Senoo, H., Nakatani, M., and Arinami, T. (2004). Association between chromogranin b gene polymorphisms and schizophrenia in the Japanese population. *Biol Psychiatry*, **56**(1), 10–17.

Ivanova, A. V., Goparaju, C. M. V., Ivanov, S. V., Nonaka, D., Cruz, C., Beck, A., Lonardo, F., Wali, A., and Pass, H. I. (2009). Protumorigenic role of HAPLN1 and its IgV domain in malignant pleural mesothelioma. *Clin Cancer Res*, **15**(8), 2602–2611.

Jandrig, B., Seitz, S., Hinzmann, B., Arnold, W., Micheel, B., Koelble, K., Siebert, R., Schwartz, A., Ruecker, K., Schlag, P. M., Scherneck, S., and Rosenthal, A. (2004). ST18 is a breast cancer tumor suppressor gene at human chromosome 8q11.2. *Oncogene*, **23**(57), 9295–9302.

Jansen, E., Ayoubi, T. A., Meulemans, S. M., and de Ven, W. J. V. (1995). Neuroendocrine-specific expression of the human prohormone convertase 1 gene. Hormonal regulation of transcription through distinct cAMP response elements. *J Biol Chem*, **270**(25), 15391–15397.

Järvinen, A.-K., Autio, R., Kilpinen, S., Saarela, M., Leivo, I., Grénman, R., Mäkitie, A. A., and Monni, O. (2008). High-resolution copy number and gene expression microarray analyses of head and neck squamous cell carcinoma cell lines of tongue and larynx. *Genes Chromosomes Cancer*, **47**(6), 500–509.

Jiang, Z., Michal, J. J., Tobey, D. J., Wang, Z., Macneil, M. D., and Magnuson, N. S. (2008). Comparative understanding of UTS2 and UTS2R genes for their involvement in type 2 diabetes mellitus. *Int J Biol Sci*, **4**(2), 96–102.

Kimura, N., Yoshida, R., ichiro Shiraishi, S., Pilichowska, M., and Ohuchi, N. (2002). Chromogranin A and chromogranin B in noninvasive and invasive breast carcinoma. *Endocr Pathol*, **13**(2), 117–122.

Klootwijk, R., Groenen, P., Schijvenaars, M., Hol, F., Hamel, B., Straatman, H., Steegers-Theunissen, R., Mariman, E., and Franke, B. (2004). Genetic variants in ZIC1, ZIC2, and ZIC3 are not major risk factors for neural tube defects in humans. *American journal of medical genetics*, **124A**(1), 40–47.

Korkola, J. E., DeVries, S., Fridlyand, J., Hwang, E. S., Estep, A. L. H., Chen, Y.-Y., Chew, K. L., Dairkee, S. H., Jensen, R. M., and Waldman, F. M. (2003). Differentiation of lobular versus ductal breast carcinomas by expression microarray analysis. *Cancer Res*, **63**(21), 7167–7175.

Kuroiwa, T., Yamamoto, N., Onda, T., and Shibahara, T. (2009). Expression of the FAM5C in tongue squamous cell carcinoma. *Oncol Rep*, **22**(5), 1005–1011.

Leja, J., Essaghir, A., Essand, M., Wester, K., Oberg, K., Tötterman, T. H., Lloyd, R., Vasmatzis, G., Demoulin, J.-B., and Giandomenico, V. (2009). Novel markers for enterochromaffin cells and gastrointestinal neuroendocrine carcinomas. *Mod Pathol*, **22**(2), 261–272.

León, R., Murray, J. I., Cragg, G., Farnell, B., West, N. R., Pace, T. C. S., Watson, P. H., Bohne, C., Boulanger, M. J., and Hof, F. (2009). Identification and characterization of binding sites on S100A7, a participant in cancer and inflammation pathways. *Biochemistry*, **48**(44), 10591–10600.

Liang, J., Xu, A., Xie, Y., Awonuga, A., and Lin, Z. (2008). Some but not all of HLA-II alleles are associated with cervical cancer in Chinese women. *Cancer Genetics and Cytogenetics*, **187**(2), 95–100.

Lux, A., Beil, C., Majety, M., Barron, S., Gallione, C. J., Kuhn, H.-M., Berg, J. N., Kioschis, P., Marchuk, D. A., and Hafner, M. (2005). Human retroviral gag- and gag-pol-like proteins interact with the transforming growth factor-beta receptor activin receptor-like kinase 1. *J Biol Chem*, **280**(9), 8482–8493.

Madeleine, M. M., Johnson, L. G., Smith, A. G., Hansen, J. A., Nisperos, B. B., Li, S., Zhao, L.-P., Daling, J. R., Schwartz, S. M., and Galloway, D. A. (2008). omprehensive analysis of HLA-A, HLA-B, HLA-C, HLA-DRB1, and HLA-DQB1 loci and squamous cell cervical cancer risk. *Cancer Res*, **68**(9), 3532–3539.

Maeda, S., Tsukada, S., Kanazawa, A., Sekine, A., Tsunoda, T., Koya, D., Maegawa, H., Kashiwagi, A., Babazono, T., Matsuda, M., Tanaka, Y., Fujioka, T., Hirose, H., Eguchi, T., Ohno, Y., Groves, C. J., Hattersley, A. T., Hitman, G. A., Walker, M., Kaku, K., Iwamoto, Y., Kawamori, R., Kikkawa, R., Kamatani, N., McCarthy, M. I., and Nakamura, Y. (2005). Genetic variations in the gene encoding TFAP2B are associated with type 2 diabetes mellitus. *J Hum Genet*, **50**(6), 283–292.

Maruo, Y., Iwai, M., Mori, A., Sato, H., and Takeuchi, Y. (2005). Polymorphism of UDP-glucuronosyltransferase and drug metabolism. *Curr Drug Metab*, **6**(2), 91–99.

Meyer, E., Lim, D., Pasha, S., Tee, L. J., Rahman, F., Yates, J. R. W., Woods, C. G., Reik, W., and Maher, E. R. (2009). Germline mutation in NLRP2 (NALP2) in a familial imprinting disorder (Beckwith-Wiedemann Syndrome). *PLoS Genet*, **5**(3), e1000423.

Monte, M., Simonatto, M., Peche, L. Y., Bublik, D. R., Gobessi, S., Pierotti, M. A., Rodolfo, M., and Schneider, C. (2006). MAGE-A tumor antigens target p53 transactivation function through histone deacetylase recruitment and confer resistance to chemotherapeutic agents. *Proc Natl Acad Sci U S A*, **103**(30), 11160–11165.

Moss, A. C., Jacobson, G. M., Walker, L. E., Blake, N. W., Marshall, E., and Coulson, J. M. (2009). SCG3 transcript in peripheral blood is a prognostic biomarker for REST-deficient small cell lung cancer. *Clin Cancer Res*, **15**(1), 274–283.

Mueller, L. P. and Machulla, H. K. G. (2002). Increased frequency of homozygosity for HLA class II loci in female patients with chronic lymphocytic leukemia. *Leuk Lymphoma*, **43**(5), 1013–1019.

Nordquist, N., Göktürk, C., Comasco, E., Eensoo, D., Merenäkk, L., Veidebaum, T., Oreland, L., and Harro, J. (2009). The transcription factor TFAP2B is associated with insulin resistance and adiposity in healthy adolescents. *Obesity (Silver Spring)*, **17**(9), 1762–1767.

Nucera, C., Eeckhoute, J., Finn, S., Carroll, J. S., Ligon, A. H., Priolo, C., Fadda, G., Toner, M., Sheils, O., Attard, M., Pontecorvi, A., Nose, V., Loda, M., and Brown, M. (2009). FOXA1 is a potential oncogene in anaplastic thyroid carcinoma. *Clin Cancer Res*, **15**(11), 3680–3689.

Pegg, A. E. (2008). Spermidine/spermine-N(1)-acetyltransferase: a key metabolic regulator. *Am J Physiol Endocrinol Metab*, **294**(6), E995–1010.

Peikert, T., Specks, U., Farver, C., Erzurum, S. C., and Comhair, S. A. A. (2006). Melanoma antigen A4 is expressed in non-small cell lung cancers and promotes apoptosis. *Cancer Res*, **66**(9), 4693–4700.

Planitzer, S. A., Machl, A. W., Rueckels, M., and Kubbies, M. (1998). Identification of a novel c-DNA overexpressed in Fanconi's anemia fibroblasts partially homologous to a putative L-3-phosphoserine-phosphatase. *Gene*, **210**(2), 297–306.

Qu, H.-Q., Marchand, L., Fréchette, R., Bacot, F., Lu, Y., and Polychronakos, C. (2007). No association of type 1 diabetes with a functional polymorphism of the LRAP gene. *Mol Immunol*, **44**(8), 2135–2138.

Quan, X., Laes, J. F., Ravoet, M., Vooren, P. V., Szpirer, J., and Szpirer, C. (2000). Localization of new, microdissection- generated, anonymous markers and of the genes Pcsk1, Dhfr, Ndub13, and Ccnb1 to rat chromosome region 2q1. *Cytogenet Cell Genet*, **88**(1-2), 119–123.

Ren, J.-G., Li, Z., and Sacks, D. B. (2007). IQGAP1 modulates activation of B-Raf. *Proc Natl Acad Sci U S A*, **104**(25), 10465–10469.

Sparks, R., Ulrich, C. M., Bigler, J., Tworoger, S. S., Yasui, Y., Rajan, K. B., Porter, P., Stanczyk, F. Z., Ballard-Barbash, R., Yuan, X., Lin, M. G., McVarish, L., Aiello, E. J., and McTiernan, A. (2004). UDP-glucuronosyltransferase and sulfotransferase polymorphisms, sex hormone concentrations, and tumor receptor status in breast cancer patients. *Breast Cancer Res*, **6**(5), R488–R498.

Stockwin, L. H., Vistica, D. T., Kenney, S., Schrump, D. S., Butcher, D. O., Raffeld, M., and Shoemaker, R. H. (2009). Gene expression profiling of alveolar soft-part sarcoma (ASPS). *BMC Cancer*, **9**, 22.

Tanabe, A., Yanagiya, T., Iida, A., Saito, S., Sekine, A., Takahashi, A., Nakamura, T., Tsunoda, T., Kamohara, S., Nakata, Y., Kotani, K., Komatsu, R., Itoh, N., Mineo, I., Wada, J., Funahashi, T., Miyazaki, S., Tokunaga, K., Hamaguchi, K., Shimada, T., Tanaka, K., Yamada, K., Hanafusa, T., Oikawa, S., Yoshimatsu, H., Sakata, T., Matsuzawa, Y., Kamatani, N., Nakamura, Y., and Hotta, K. (2007). Functional single-nucleotide polymorphisms in the secretogranin III (SCG3) gene that form secretory granules with appetite-related neuropeptides are associated with obesity. *J Clin Endocrinol Metab*, **92**(3), 1145–1154.

Taylor, G. M., Dearden, S., Payne, N., Ayres, M., Gokhale, D. A., Birch, J. M., Blair, V., Stevens, R. F., Will, A. M., and Eden, O. B. (1998). Evidence that an HLA-DQA1-DQB1 haplotype influences susceptibility to childhood common acute lymphoblastic leukaemia in boys provides further support for an infection-related aetiology. *Br J Cancer*, **78**(5), 561–565.

Thomassen, M., Tan, Q., and Kruse, T. A. (2009). Gene expression meta-analysis identifies chromosomal regions and candidate genes involved in breast cancer metastasis. *Breast Cancer Res Treat*, **113**(2), 239–249.

Torre, D. L., Maugeri, R., Angileri, F. F., Pezzino, G., Conti, A., Cardali, S. M., Calisto, A., Sciarrone, G., Misefari, A., Germanò, A., and Tomasello, F. (2009). Human leukocyte antigen frequency in human high-grade gliomas: a case-control study in Sicily. *Neurosurgery*, **64**(6), 1082–8; discussion 1088–9.

Uemura, M. and Takeichi, M. (2006). Alpha N-catenin deficiency causes defects in axon migration and nuclear organization in restricted regions of the mouse brain. *Dev Dyn*, **235**(9), 2559–2566.

Vaquero-Lorenzo, C., Bermudo-Soriano, C. R., Perez-Rodriguez, M. M., Diaz-Hernandez, M., López-Castromán, J., Fernandez-Piqueras, J., Saiz-Ruiz, J., and Baca-Garcia, E. (2009). Positive association between SAT-1 -1415T/C polymorphism and anxiety. *Am J Med Genet B Neuropsychiatr Genet*, **150B**(4), 515–519.

Wang, C., Xiao, Y., Hu, Z., Chen, Y., Liu, N., and Hu, G. (2008). PEG10 directly regulated by E2Fs might have a role in the development of hepatocellular carcinoma. *FEBS Lett*, **582**(18), 2793–2798.

Wang, L. J., Jin, H. C., Wang, X., Lam, E. K. Y., Zhang, J. B., Liu, X., Chan, F. K. L., Si, J. M., and Sung, J. J. Y. (2009). ZIC1 is downregulated through promoter hypermethylation in gastric cancer. *Biochem Biophys Res Commun*, **379**(4), 959–963.

Wendland, J. R., Moya, P. R., Timpano, K. R., Anavitarte, A. P., Kruse, M. R., Wheaton, M. G., Ren-Patterson, R. F., and Murphy, D. L. (2009). A haplotype containing quantitative trait loci for SLC1A1 gene expression and its association with obsessive-compulsive disorder. *Arch Gen Psychiatry*, **66**(4), 408–416.

Wenyi, Z., Suzuki, S., Hirai, M., Hinokio, Y., Tanizawa, Y., Matsutani, A., Satoh, J., and Oka, Y. (2003). Role of urotensin II gene in genetic susceptibility to Type 2 diabetes mellitus in Japanese subjects. *Diabetologia*, **46**(7), 972–976.

Westerman, B. A., Neijenhuis, S., Poutsma, A., Steenbergen, R. D. M., Breuer, R. H. J., Egging, M., van Wijk, I. J., and Oudejans, C. B. M. (2002). Quantitative reverse transcription-polymerase chain reaction measurement of HASH1 (ASCL1), a marker for small cell lung carcinomas with neuroendocrine features. *Clin Cancer Res*, **8**(4), 1082–1086.

White, C. D., Brown, M. D., and Sacks, D. B. (2009). IQGAPs in cancer: a family of scaffold proteins underlying tumorigenesis. *FEBS Lett*, **583**(12), 1817–1824.

Wong, M. P., Cheang, M., Yorida, E., Coldman, A., Gilks, C. B., Huntsman, D., and Berean, K. (2008). Loss of desmoglein 1 expression associated with worse prognosis in head and neck squamous cell carcinoma patients. *Pathology*, **40**(6), 611–616.

Wong, Y. F., Cheung, T. H., Lo, K. W. K., Yim, S. F., Siu, N. S. S., Chan, S. C. S., Ho, T. W. F., Wong, K. W. Y., Yu, M. Y., Wang, V. W., Li, C., Gardner, G. J., Bonome, T., Johnson, W. B., Smith, D. I., Chung, T. K. H., and Birrer, M. J. (2007). Identification of molecular markers and signaling pathway in endometrial cancer in Hong Kong Chinese women by genome-wide gene expression profiling. *Oncogene*, **26**(13), 1971–1982.

Yang, J., Siqueira, M. F., Behl, Y., Alikhani, M., and Graves, D. T. (2008). The transcription factor ST18 regulates proapoptotic and proinflammatory gene expression in fibroblasts. *FASEB J*, **22**(11), 3956–3967.

Yi, L., Gu, Y. H., Wang, X. L., An, L. Z., Xie, X. D., Shao, W., Ma, L. Y., Fang, J. R., An, Y. D., Wang, F., and Zhang, D. L. (2006). Association of ACE, ACE2 and UTS2 polymorphisms with essential hypertension in Han and Dongxiang populations from north-western China. *J Int Med Res*, **34**(3), 272–283.

Zafrakas, M., Petschke, B., Donner, A., Fritzsche, F., Kristiansen, G., Knüchel, R., and Dahl, E. (2006). Expression analysis of mammaglobin A (SCGB2A2) and lipophilin B (SCGB1D2) in more than 300 human tumors and matching normal tissues reveals their co-expression in gynecologic malignancies. *BMC Cancer*, **6**, 88.

Zhang, H., Rakha, E., Ball, G., Spiteri, I., Aleskandarany, M., Paish, E., Powe, D., Macmillan, R., Caldas, C., Ellis, I., and Green, A. (2009). The proteins FABP7 and OATP2 are associated with the basal phenotype and patient outcome in human breast cancer. *Breast Cancer Res Treat*.

Zhou, L., Zhang, R., Yao, W., Wang, J., Qian, A., Qiao, M., Zhang, Y., and Yuan, Y. (2009). Decreased expression of angiotensin-converting enzyme 2 in pancreatic ductal adenocarcinoma is associated with tumor progression. *Tohoku J Exp Med*, **217**(2), 123–131.
